# Genetic characteristics and phylogenetic analysis of three Chinese ethnic groups using the Huaxia Platinum System

**DOI:** 10.1038/s41598-018-20871-7

**Published:** 2018-02-05

**Authors:** Mengge Wang, Zheng Wang, Guanglin He, Zhenjun Jia, Jing Liu, Yiping Hou

**Affiliations:** 1 Institute of Forensic Medicine, West China School of Basic Science and Forensic Medicine, Sichuan University, Chengdu, 610041 China; 2 Department of Criminal Science and Technology, People’s Public Security University of China, Beijing, 100038 China

## Abstract

Short tandem repeats (STRs) are attractive to genetic applications like forensic, anthropological and population genetics studies. The Huaxia Platinum System was specifically developed to allow co-amplification and detection of all markers in the expanded CODIS core loci and the Chinese National Database. In this study, in continuation to our previous validation study, 568 unrelated individuals were firstly genotyped to investigate the effectiveness of this novel assay in 3 main ethnic groups of China (Han, Tibetan and Yi). The combined power of discrimination (CPD) were 0.9999999999999999999999999992, 0.999999999999999999999999992, 0.999999999999999999999999998, respectively, and the combined power of exclusion (CPE) were 0.9999999999, 0.999999995, 0.999999998, respectively. Next, genetic relationships along administrative and ethnic divisions were analyzed using pairwise genetic distances, multidimensional scaling (MDS), principal component analysis (PCA) and phylogenetic analysis. The Han ethnicity showed a high genetic homogeneity all across China, and significant genetic differences existed between Han groups and some minority groups, most prominently for the Tibetans, the Uyghurs, the Kazakhs, the Miaos, the Zhuangs and the Dais. Aforementioned results suggested that the Huaxia Platinum System is polymorphic and informative, which provides an efficient tool not only for human forensics, but also for population genetics studies.

## Introduction

Short tandem repeats (STRs) are DNA fragments with a variable number of tandemly repeated short (2–6 bp) sequence motifs, such as (GATA)n^1^. STR genotyping has now been applied in various aspects of human identification in forensic investigations for nearly 30 years^2,3^. Recently, to minimize adventitious matches and to facilitate data sharing, the Combined DNA Index System (CODIS) was upgraded from 13 loci to 20 loci (i.e. the expanded CODIS)^4^, while a parallel process occurred in China where 20 loci are required for uploading DNA profiles to the Chinese National Database (CND), the world’s biggest DNA databases^5,6^. However, 6 non-overlapped loci exist between the expanded CODIS and the CND^6,7^. The Huaxia Platinum System (Thermo Fisher Scientific, MA, USA) is a 25-locus, six-dye, multiplex that allows co-amplification and fluorescent detection of the 23 autosomal loci (D1S1656, D2S1338, D2S441, D3S1358, D5S818, D7S820, D8S1179, D10S1248, D12S391, D13S317, D16S539, D18S51, D19S433, D21S11, D22S1045, D6S1043, CSF1PO, FGA, TH01, TPOX, VWA, Penta D and Penta E) including all the recommended core loci in the expanded CODIS and the CND as well as Amelogenin and Y-InDel (rs2032678) for gender determination^8^. Our previous validation study demonstrated that this novel assay is robust, sensitive, specific and reliable, and forensic parameters is polymorphic and informative in three main ethnic groups of China, Sichuan Han, Xinjiang Uygur and Tibet Tibetan^7^.

China, the world’s most populous country and the world’s second-largest state by land area, emerged as one of the world’s earliest civilizations in the fertile basin of the Yellow River in the North China Plain. The nation officially recognizes 56 distinct ethnic groups, widely disseminated in 34 administrative regions, and the population substructure is further complicated^9,10^. The Han population, accounting for 92% of the total population in China, is widely distributed in mainland territory of China. The Yi people are a typical ethnic minority in China and the largest ethnic minority group in Sichuan Liangshan Yi Autonomous Prefecture. Most of them live in mountainous regions and often carve out their existence on the sides of steep mountain slopes. The Tibetan is one of the oldest peoples in China and South Asia and Tibetan population mainly reside throughout the Qinghai-Tibetan Plateau for hundreds of generations, and has genetic adaptations of distinct combinations of phenotype in high-altitude (>4000 m). With economic growth and traffic development, some Tibetans began to migrate to plain areas. Sichuan is home to a large community of Tibetans, with 30,000 permanent Tibetan residents and up to 200,000 Tibetan floating population.

In continuation to our previous studies^7,11^, the present study characterizes the genetic diversity of the Huaxia Platinum System in 3 main ethnic groups of China (193 Hainan Hans, 198 Sichuan Tibetans and 177 Sichuan Yis). Additionally, genetic data of our present investigated individuals and other previously studied 56 Chinese populations^7,11–44^ were used to investigate genetic relationships along administrative and ethnic divisions.

## Results and Discussion

### Genetic parameters of the Huaxia Platinum System

Hainan, separated by the Qiongzhou Strait from the Leizhou Peninsula of Guangdong, is the smallest and southernmost province of China. The population density of Hainan is low compared to most Chinese coastal provinces. This study was carried out to provide the first batch of 23 STRs data of Hainan Han population (193 unrelated individuals) using the Huaxia Platinum System. Meanwhile, we continue to evaluate the forensic efficiency of this novel assay for application in 2 main ethnic groups in Sichuan. Sichuan consists of two geographically distinct parts: the eastern part is mostly within the fertile Sichuan basin and the western part consists of the numerous mountain range. Han Chinese, the majority of the province’s population, mainly reside in the eastern portion, while significant minorities of Yi and Tibetan people reside in the western portion that are impacted by inclement weather and natural disasters. Sichuan Han have been investigated in our previous studies^11^, in the present study Sichuan Yi (177 unrelated individuals) and Tibetan people (198 unrelated individuals) were analyzed.

No significant deviation from Hardy-Weinberg disequilibrium was observed (Table 1) and no significant deviations from linkage disequilibrium between pairwise STR loci after Bonferroni correction in 3 main ethnic groups (Supplementary Tables 1–3). The allele frequency distributions are listed in Supplementary Tables 4–6 and forensic parameters including observed heterozygosity (Ho), expected heterozygosity (He), power of discrimination (PD), power of exclusion (PE) and typical paternity index (TPI) for each locus are presented in Table 1.

**Table 1:** Forensic parameters for 23 autosomal STR loci of the Huaxia Platinum system in three Chinese ethnic groups.

Populations	FP	D3S1358	vWA	D16S539	CSF1PO	TPOX	D8S1179	D21S11	D18S51	Penta E	D2S441	D19S433	TH01
Han	PD	0.8594	0.9176	0.9108	0.8849	0.8028	0.9525	0.9404	0.9574	0.9840	0.9034	0.9486	0.8605
	PE	0.4519	0.5762	0.6740	0.4686	0.2290	0.6944	0.7149	0.7460	0.8411	0.5390	0.6440	0.4039
	TPI	1.7545	2.3537	3.1129	1.8208	1.0966	3.3276	3.5741	4.0208	6.4333	2.1444	2.8382	1.5820
	Ho	0.7165	0.7887	0.8402	0.7268	0.5412	0.8505	0.8608	0.8763	0.9227	0.7680	0.8247	0.6856
	He	0.7178	0.7894	0.7845	0.7414	0.6233	0.8486	0.8253	0.8583	0.9233	0.7614	0.8281	0.6939
	P	0.0779	0.5427	0.4334	0.3094	0.0021	0.1146	0.5211	0.1915	0.7198	0.0916	0.9417	0.0209
Yi	PD	0.8374	0.9216	0.9255	0.8577	0.7927	0.9458	0.9486	0.9596	0.9794	0.9321	0.9410	0.8411
	PE	0.4317	0.5440	0.5440	0.4145	0.3894	0.6371	0.7475	0.6696	0.6587	0.5050	0.6587	0.3060
	TPI	1.6792	2.1707	2.1707	1.6182	1.5345	2.7813	4.0455	3.0690	2.9667	1.9778	2.9667	1.2899
	Ho	0.7006	0.7684	0.7684	0.6893	0.6780	0.8192	0.8757	0.8362	0.8305	0.7458	0.8305	0.6158
	He	0.6892	0.8010	0.7977	0.7006	0.6327	0.8333	0.8394	0.8488	0.9111	0.7977	0.8232	0.6635
	P	0.3459	0.2120	0.2491	0.6302	0.1564	0.2955	0.9337	0.9654	0.0011	0.1947	0.4908	0.2176
Tibetan	PD	0.8523	0.9239	0.8979	0.8851	0.7677	0.9409	0.9422	0.9284	0.9841	0.9013	0.9494	0.8367
	PE	0.3260	0.5879	0.4739	0.4416	0.2227	0.5695	0.6349	0.6349	0.6737	0.5337	0.6254	0.3810
	TPI	1.3446	2.4268	1.8426	1.7155	1.0815	2.3140	2.7639	2.7639	3.1094	2.1170	2.6892	1.5076
	Ho	0.6263	0.7929	0.7273	0.7071	0.5404	0.7828	0.8182	0.8232	0.8384	0.7677	0.8131	0.6717
	He	0.6877	0.7970	0.7486	0.7381	0.5878	0.8180	0.8286	0.8083	0.9207	0.7564	0.8344	0.6584
	p	0.1167	0.4993	0.2084	0.4716	0.0636	0.4067	0.2367	0.0195	0.0853	0.6143	0.3738	0.9015
**Populations**	**FP**	**FGA**	**D22S1045**	**D5S818**	**D13S317**	**D7S820**	**D6S1043**	**D10S1248**	**D1S1656**	**D12S391**	**D2S1338**	**Penta D**	
Han	PD	0.9668	0.9116	0.9208	0.9330	0.9084	0.9666	0.9067	0.9514	0.9540	0.9635	0.9231	
	PE	0.6640	0.5482	0.6243	0.4857	0.5299	0.7669	0.5209	0.6640	0.7149	0.7564	0.5952	
	TPI	3.0156	2.1932	2.6806	1.8922	2.0978	4.3864	2.0532	3.0156	3.5741	4.1957	2.4744	
	Ho	0.8351	0.7732	0.8144	0.7371	0.7629	0.8866	0.7577	0.8351	0.8608	0.8814	0.7938	
	He	0.8692	0.7759	0.7917	0.8055	0.7689	0.8744	0.7650	0.8435	0.8527	0.8708	0.7756	
	P	0.2273	0.9367	0.9618	0.0675	0.4617	0.4772	0.8349	0.0951	0.1252	0.3016	0.4459	
Yi	PD	0.9579	0.8988	0.9124	0.9376	0.9280	0.9693	0.8984	0.9410	0.9535	0.9580	0.9275	
	PE	0.5949	0.5743	0.5641	0.5341	0.6158	0.6587	0.5641	0.5845	0.6478	0.6587	0.6805	
	TPI	2.4722	2.3421	2.2821	2.1190	2.6176	2.9667	2.2821	2.4054	2.8710	2.9667	3.1786	
	Ho	0.8023	0.7853	0.7853	0.7627	0.8079	0.8305	0.7797	0.7966	0.8249	0.8305	0.8418	
	He	0.8545	0.7650	0.7802	0.8118	0.8033	0.8816	0.7590	0.8233	0.8461	0.8547	0.7971	
	P	0.0277	0.9585	0.3853	0.3815	0.5358	0.0039	0.4065	0.3372	0.5038	0.2368	0.7628	
Tibetan	PD	0.9598	0.8888	0.9100	0.9278	0.9219	0.9684	0.8904	0.9360	0.9478	0.9517	0.9360	
	PE	0.6835	0.5249	0.4906	0.7333	0.5695	0.6934	0.5076	0.4906	0.6737	0.6047	0.5695	
	TPI	3.2097	2.0729	1.9135	3.8269	2.3140	3.3167	1.9900	1.9135	3.1094	2.5385	2.3140	
	Ho	0.8434	0.7626	0.7374	0.8687	0.7879	0.8485	0.7525	0.7424	0.8384	0.8081	0.7828	
	He	0.8595	0.7520	0.7747	0.8121	0.7929	0.8762	0.7536	0.8045	0.8318	0.8438	0.8079	
	p	0.5043	0.3654	0.0517	0.2692	0.3571	0.5300	0.1177	0.1855	0.3064	0.0100	0.7958	

FP: forensic parameters; PD: power of discrimination; PIC: polymorphism information content; PE: probability of exclusion; TPI: Typical Paternity Index; Ho: observed heterozygosity; He: expected heterozygosity; p: probability values of exact tests for Hardy–Weinberg equilibrium.

A total of 246 alleles were observed in Hainan Hans with corresponding allele frequencies ranging from 0.0026 to 0.5207. The PD, PE, TPI, Ho, He varied from 0.8028 to 0.9840, 0.2290 to 0.8411, 1.0966 to 6.4333, 0.5412 to 0.9227 and 0.6233 to 0.9233, respectively. The combined power of discrimination (CPD), combined power of exclusion (CPE) are 0.9999999999999999999999999992 and 0.9999999999. For Sichuan Yi population, a total of 245 alleles were identified with corresponding allele frequencies varied from 0.0028 to 0.5057. The values of PD, PE, TPI, Ho, He spanned from 0.7927 to 0.9794, 0.3060 to 0.7475, 1.2899 to 4.0455, 0.6158 to 0.8757 and 0.6327 to 0.9111, respectively. The CPD, CPE are 0.999999999999999999999999998 and 0.999999998. For Sichuan Tibetan population, a total of 231 alleles were detected with corresponding allele frequency varied from 0.0025 to 0.5808. The PD, PE, TPI, Ho, He spanned from 0.7677 to 0.9841, 0.2227 to 0.7333, 1.0815 to 3.8269, 0.5404 to 0.8687 and 0.5878 to 0.9207, respectively. The values of CPD and CPE are 0.999999999999999999999999992 and 0.999999995. Meanwhile, the CPD and CPE based on the loci covered by the PowerPlex 21 System, Golden^TM^ DNA ID system 20 A kit and AGCU EX22 kit were estimated separately and listed in Supplementary Table 7. Obviously, the above-mentioned forensic parameters of these 23 loci demonstrated that the Huaxia Platinum multiplex system is a more informative and discriminative system as required for forensic DNA genotyping and databasing.

### Population pairwise differences

The different nationalities of China were widely distributed in 34 administrative divisions, and the population substructure was complicated. In order to illuminate the genetic affinity among different nationalities, population comparisons were performed between our three studied groups and 56 previously investigated populations (40 Han populations, 15 ethnic minorities as well as Vietnamese of Yunnan). The Locus-by-Locus F_st_ and corresponding p values showed that statistically significant differences were observed between Hainan Han and Yunnan Miao at 11 loci, between Yunnan Vietnamese at 6 loci, and between Xinjiang Uyghur at 4 loci after Bonferroni adjustment (p < 0.00004) (Supplementary Table 8). As showed in Supplementary Tables 9–10, after Bonferroni correction (p < 0.00004), statistically significant genetic differentiations were found between Sichuan Yi and Yunnan Miao at 10 loci, between Yunnan Vietnamese at 7 loci, and between Inner Mongolia at 4 loci. Genetic differences were existed between Sichuan Tibetan and Yunnan Miao at 10 loci, between Yunnan Vietnamese at 9 loci, and between Taiwan Han at 3 loci.

### Principal component analysis

**Figure 1:**
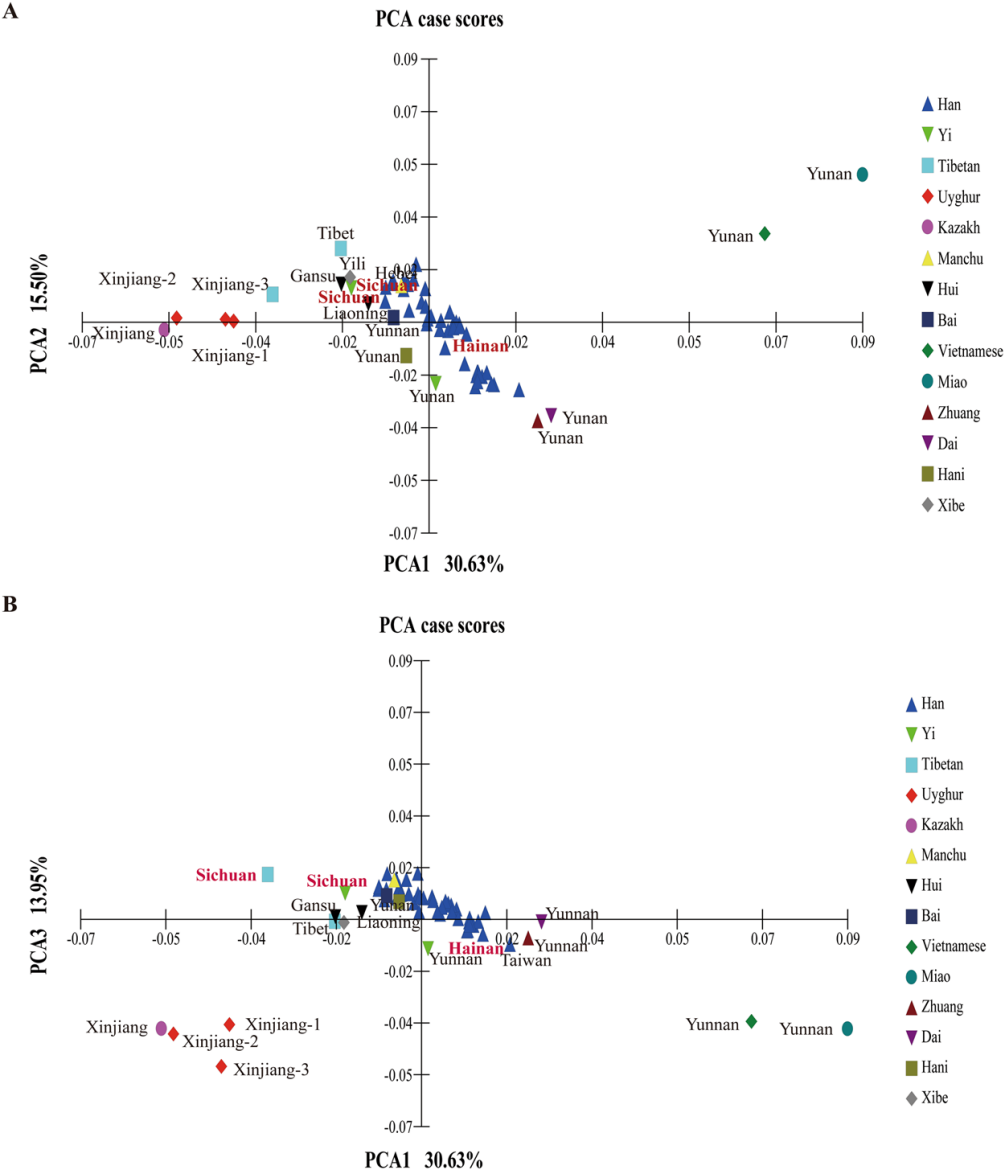
Principal component analysis based on 19 overlapped STR loci of our studied populations (bold and red) and 46 reference Chinese Han populations.

Principal component analysis among the 59 groups was carried out been prepared on the basis of allele frequency distributions of 19 STR loci. As displayed in Fig. 1, the first principal component, the second principal component and the third principal component account for 30.63%, 15.50%, and 13.95% of the total variance, respectively, which can clearly differentiate the Tibetan, Uyghur, Kazakh, Miao and Vietnamese with other groups, but Han Chinese populations residing in different administrative regions were conglomerated together, which revealed that the genetic similarity was widely existed among Han Chinese populations distributed in different administrative divisions.

### Multidimensional scaling analysis

To further explore the genetic diversity and phylogenetic characteristic among Chinese populations, Nei’s standard genetic distances were calculated among 59 Chinese populations and presented in Supplementary Table 11. The largest genetic distances were observed between Miao and our three investigated groups: Hainan Han, Sichuan Yi, Sichuan Tibetan (R_st_ = 0.0940, R_st_ = 0.1103 and R_st_ = 0.1442, respectively), while Guangzhou Han is the most closely related to Hainan Han (Rst = 0.0107), Shanghai Han is the most closely related to Sichuan Yi (R_st_ = 0.0116), and Yunnan Bai is the closest to Sichuan Tibetan (R_st_ = 0.0199).

**Figure 2:**
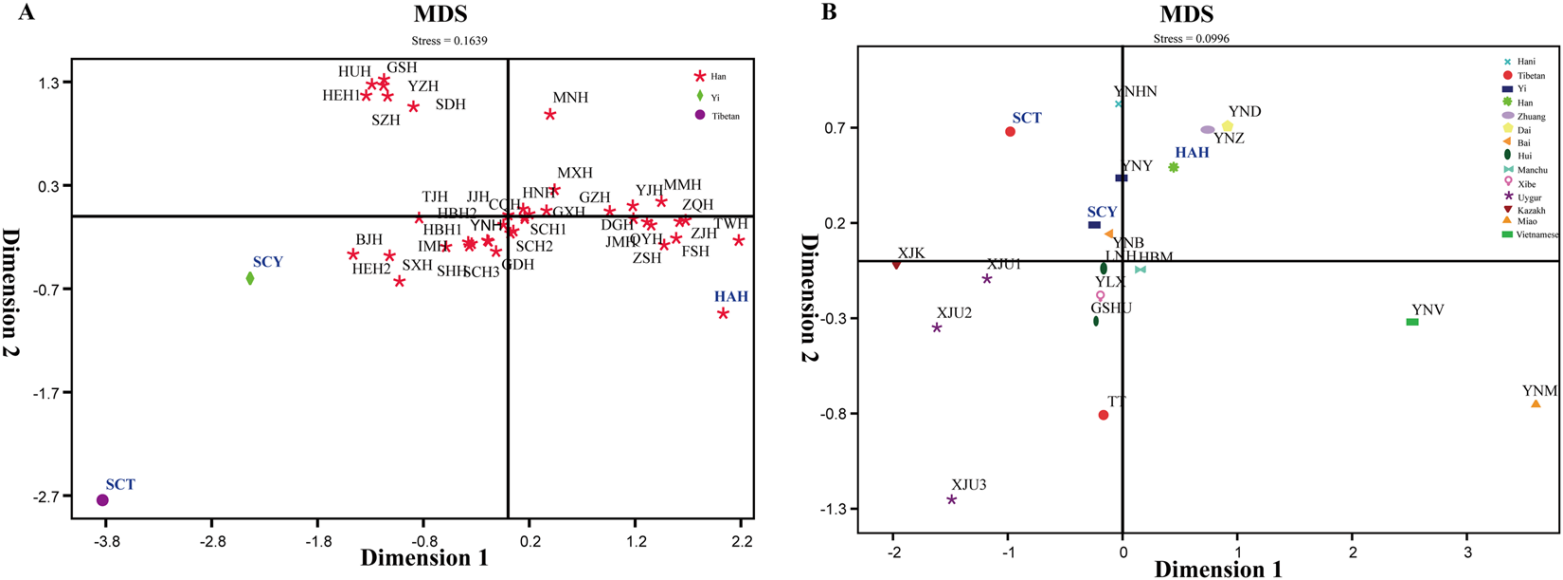
Multidimensional Scaling plots (MDS) constructed based on Nei’s genetic distances calculated by allele frequency distributions of 19 overlapped autosomal STRs. (**A**) MDS of our studied populations (bold and blue) and 40 reference Chinese Han populations (the information of abbreviations are presented in Supplementary Table 12). (**B**) MDS of our studied populations (bold and blue) and 16 reference ethnic minorities (the information of abbreviations are presented in Supplementary Table 12).

Furthermore, evolutionary relationships among 59 Chinese populations were inferred from MDS (Supplementary Fig. 1) on the basis of genetic distance matrix. As shown in Supplementary Fig. 1, 12 out of 59 populations (3 Xinjiang Uyghurs, Xinjiang Kazakh, Sichuan Tibetan, Tibet Tibetan, Yunnan Miao, Yunnan Vietnamese, Yunnan Dai, Yunnan Zhuang, Yunnan Yi, Yunnan Hani) were isolated and fall into the surrounding of MDS plots, and other populations were clustered together. For the sake of further ascertaining the genetic differentiation between our three investigated populations with the 40 reference Han populations, and with previously reported ethnic minorities, the other two MDS scatter diagrams were illustrated based on genetic distances values (Fig. 2).

As shown in Fig. 2A, genetic divergence has existed between the two studied ethnic minorities Sichuan Yi, Sichuan Tibetan and Chinese Han populations distributed in different administrative regions obviously, and subtle differentiation was found between the Hainan Han and other Han Chinese populations. The findings were in line with the results of PCA. The visualization of Nei’s genetic distances values between our investigated nationalities and reference ethnic minority groups (Fig. 2B) demonstrated that there were significant differences among different minorities. Additionally, our research objects Sichuan Tibetan, Hainan Han clearly separated with other groups, the Sichuan Yi clustered with Yunan Bai.

### Phylogenetic relationship analysis

**Figure 3:**
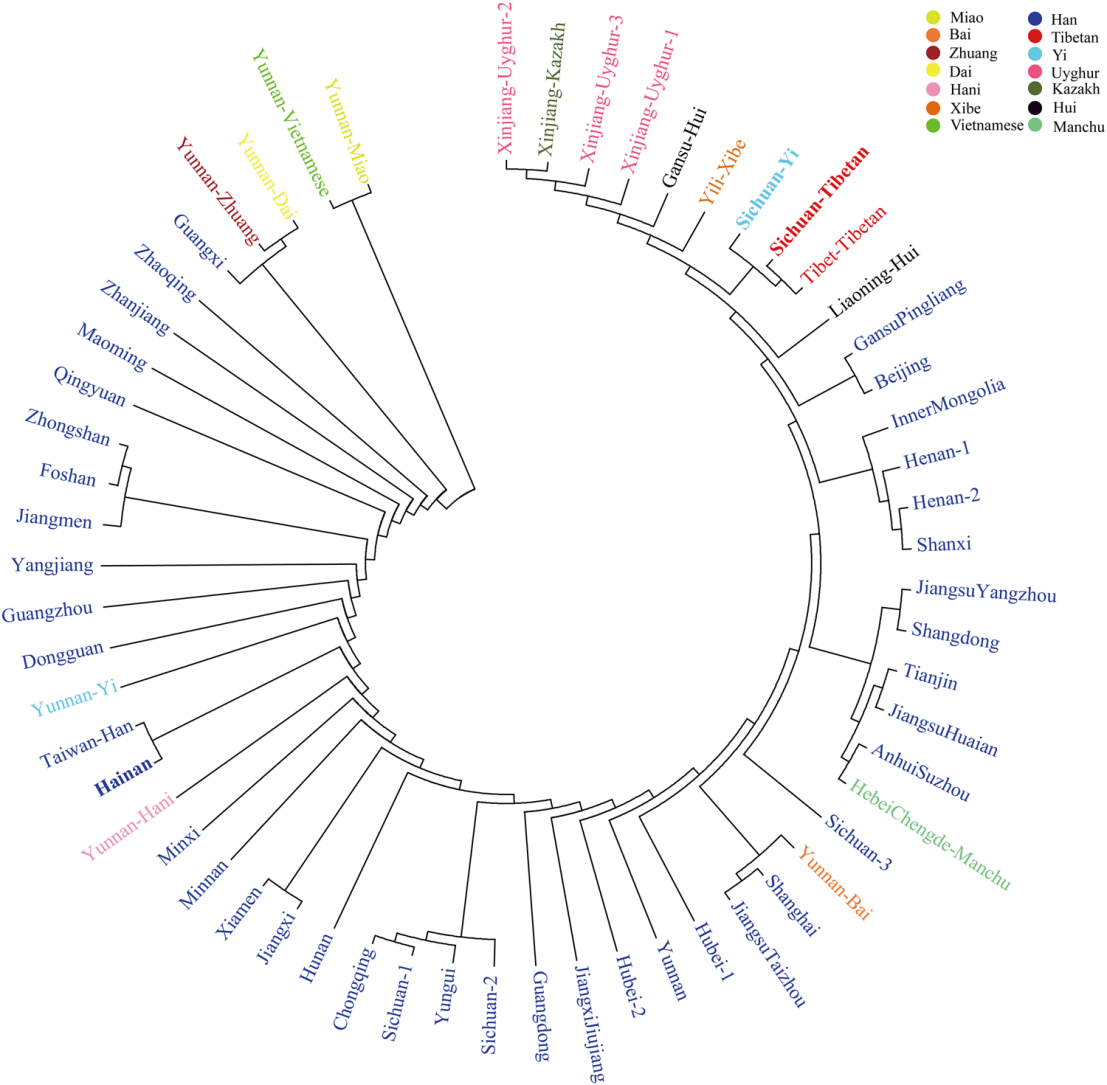
Phylogenetic tree among three studied populations (red and bold) and 56 reference populations. Phylogenetic tree was constructed by the Neighbor-Joining method based on 19 overlapped STR loci in the Mega 7.0 software.

To further explore the phylogenetic characteristics among Chinese populations, a phylogenetic tree was constructed using the neighbor-joining method. In the dendrogram (Fig. 3), two main clusters were clearly identified: one consisted of Yunnan Miao and Yunnan Vietnamese, and the other comprised 57 populations clustered together. Our investigated Hainan Han grouped with geographically ethnically close population Taiwan Han, Sichuan Tibetan first clustered with Tibet Tibetan and then clustered with Sichuan Yi. The phylogenetic structure revealed by Nei’s genetic distance matrix was in conformity with the characteristics revealed by PCA and MDS, which also in line with the results obtained in our previous researches based on Y-Chromosomal and X-Chromosomal genetic markers^45–47^.

To make a comprehensive population comparison based on autosomal genetic markers, we investigated the genetic variations in 568 unrelated individuals by using 23 autosomal STR loci and explored genetic relationships among 59 Chinese groups distributed in different administrative regions. Genetic data presented here provide basic information on the ethnic and geographical population differentiation required by the forensic genetics. Genetic differences within ethnicities were usually of minor magnitude, especially the Han nationality, which, despite their large sample size, showed a prominent genetic homogeneity. In this study, a genetic distinction between Northern and Southern Han^48^ or a North-South gradient genetic difference^10^ based on Y chromosomal genetic data has not been observed. It’s explicable that Y chromosome has the features of without recombination between loci and isolation-by-distance model, and in modern times, huge migrations of the Han might have further contributed to a homogeneity of the genetic landscape of China. We observed substantial genetic divergences among some ethnic groups, most notably Tibetans, Uyghurs, Kazakh, Miao, Vietnamese and Dai, and most other ethnicities. It’s explainable by different ancestries as well as special geographical and cultural background. Moreover, in order to provide more information for population genetics studies, more in-depth statistical analysis of our genetic data and larger sample sizes of some ethnicities are needed in future studies.

## Conclusions

In summary, we provided the first batch of genetic polymorphism data of Hainan Han, Sichuan Yi and Sichuan Tibetan using 23 autosomal STR loci included in the Huaxia Platinum System. The results of forensic characteristics demonstrated that this new 25-plex multiplex system is highly polymorphic and informative in the studied populations and can be employed as a powerful tool for forensic applications. The inter-population comparisons, PCA, MDS and phylogenetic analysis manifested that no significant genetic distinction was found between northern and southern Han Chinese populations, but subtle divergence was observed between Hainan Han and other Han populations. And the inter-population comparisons, PCA, MDS and phylogenetic analysis consistently demonstrated that significant genetic differentiation was existed between minority ethnic groups (particularly in Sichuan Tibetan, Tibet Tibetan, Xinjiang Uyghur, Xinjiang Kazakh, Yunnan Miao, Yunnan Vietnamese, Yunnan Zhuang and Yunnan Dai) and Han populations. The results of genetic population substructure pattern can happen for the reasons of large-scale population migration, ethnic intermarriage, random mating and gene flow among different ethnicities or one nationality from distinct geographic regions.

## Methods

### Ethics Statement

Human blood samples were collected upon approval of the Ethics Committee at the Institute of Forensic Medicine, Sichuan University. Written informed consent was obtained from each participant. All the methods were carried out in accordance with the approved guidelines of Institute of Forensic Medicine, Sichuan University. This study was approved by the Ethics Committee of Sichuan University (Approval Number: K2015008).

### Sample preparation

568 peripheral blood samples were collected from 193 unrelated Han Chinese recruited from Hainan Province, 177 unrelated Yi Chinese recruited from Sichuan Liangshan Yi Autonomous Prefecture and 198 Tibetan Chinese recruited from Sichuan Province.

Human genomic DNA was extracted using the Purelink Genomic DNA Mini Kit (Thermo Fisher Scientific) according to the manufacturer’s instructions. The quantity of the DNA template was determined using Quantifiler Human DNA Quantification Kit (Thermo Fisher Scientific) on a 7500 Real-time PCR System (Thermo Fisher Scientific). DNA samples were then normalized to 1.0 ng/μl and stored at −20 °C until amplification.

### Amplification and genotyping

PCR amplification was performed with 27 PCR cycles in a ProFlex PCR System (Thermo Fisher Scientific) following the manufacturer’s protocol. Amplification products were separated and detected on an Applied Biosystems 3500 Genetic Analyzers using POP-4 polymer and 36 cm capillary array according to the manufacturer’s recommendations. Allele allocation was carried out with GeneMapper ID-X v.1.4 software (Thermo Fisher Scientific) using the allelic ladder and the set of bins and panels provided by the manufacturer.

### Population studies

To evaluate the forensic efficiency of this novel STR system for application in 3 main ethnic groups of China, genotype data of 568 unrelated individuals including 193 Han, 177 Yi and 198 Tibetan were analyzed. Population indices including allele frequency, heterozygosity, Hardy–Weinberg equilibrium (HWE) and the possible presence of linkage disequilibrium (LD) among loci pairs were obtained using Arlequin software v3.5.2.2^49^. Forensic parameters were estimated by calculating power of discrimination (PD), power of exclusion (PE) and typical paternity index (TPI) using modified PowerStats V12 spreadsheet (Promega)^50^.

Furthermore, to further investigate the phylogenetic relationships among Chinese populations, a comprehensive population comparison among 59 groups^7,11–44^ was conducted using Locus-by-Locus comparisons (F_st_) based on 19 overlapping STR loci (D2S1338, D3S1358, D5S818, D6S1043, D7S820, D8S1179, D12S391, D13S317, D16S539, D18S51, D19S433, D21S11, CSF1PO, FGA, Penta D, Penta E, TH01, TPOX, VWA) following Slatkin’s linearized F_st_^51^. The pairwise F_st_’s can be used as short-term genetic distances between populations, with the application of a slight transformation to linearize the distance with population divergence time. The detailed information and abbreviations of aforementioned populations are shown in Supplementary Table 12. The Principal component analysis scatter plot was depicted by MVSP v3.22 software^52^ and multidimensional scaling analysis (MDS) was conducted in SPSS software (IBM SPSS, version 19.0, Chicago). Unbiased estimate of Nei’s standard pairwise genetic distance was calculated using the Phylip3.695 package. A neighbor-joining phylogenetic tree was delineated in the Molecular Evolutionary Genetics Analysis 7.0 (MEGA 7.0) software^53^.

### Quality control

Control DNA 007 (Thermo Fisher Scientific) and ddH_2_O were used as positive and negative controls respectively for each batch of amplification and genotyping. All experiments were conducted at the Forensic Genetics Laboratory of Institute of Forensic Medicine, Sichuan University, which is an accredited laboratory (ISO 17025), in accordance with quality control measures. Additionally, the laboratory has been accredited by the China National Accreditation Service for Conformity Assessment (CNAS).

## Electronic supplementary material

Supplementary Figure S1 and Supplementary Tables S1-12
