# Supplementary material for: Genetic characteristics and phylogenetic analysis of three Chinese ethnic groups using the Huaxia Platinum System
Source: Sci Rep. 2018 Feb 5;8:2429. doi: 10.1038/s41598-018-20871-7 (PMC5799253; doi:10.1038/s41598-018-20871-7)
Supplement: Supplementary file 1 — Supplementary Figure S1 and Supplementary Tables S1-12 [file 41598_2018_20871_MOESM1_ESM.pdf]

Supplementary Figure S1 and Supplementary Tables S1-S12

Genetic characteristics and phylogenetic analysis of three Chinese ethnic groups using the Huaxia Platinum System

Mengge Wang[1]*, Zheng Wang[1]*, Guanglin He[1], Zhenjun Jia[2], Jing Liu[1], Yiping Hou[1#]

[1] Institute of Forensic Medicine, West China School of Basic Science and Forensic Medicine, Sichuan University, Chengdu 610041, China

[2] Department of Criminal Science and Technology, People's Public Security University of China, Beijing 100038, China

* These authors contributed equally to this work

[#] Corresponding author:

Yiping Hou

Institute of Forensic Medicine, West China School of Basic Science and Forensic Medicine, Sichuan University, Chengdu 610041, China

Phone: +86-28-85501550; Fax: +86-28-85501549.

Email address: profhou@yahoo.com, forensic@scu.edu.cn

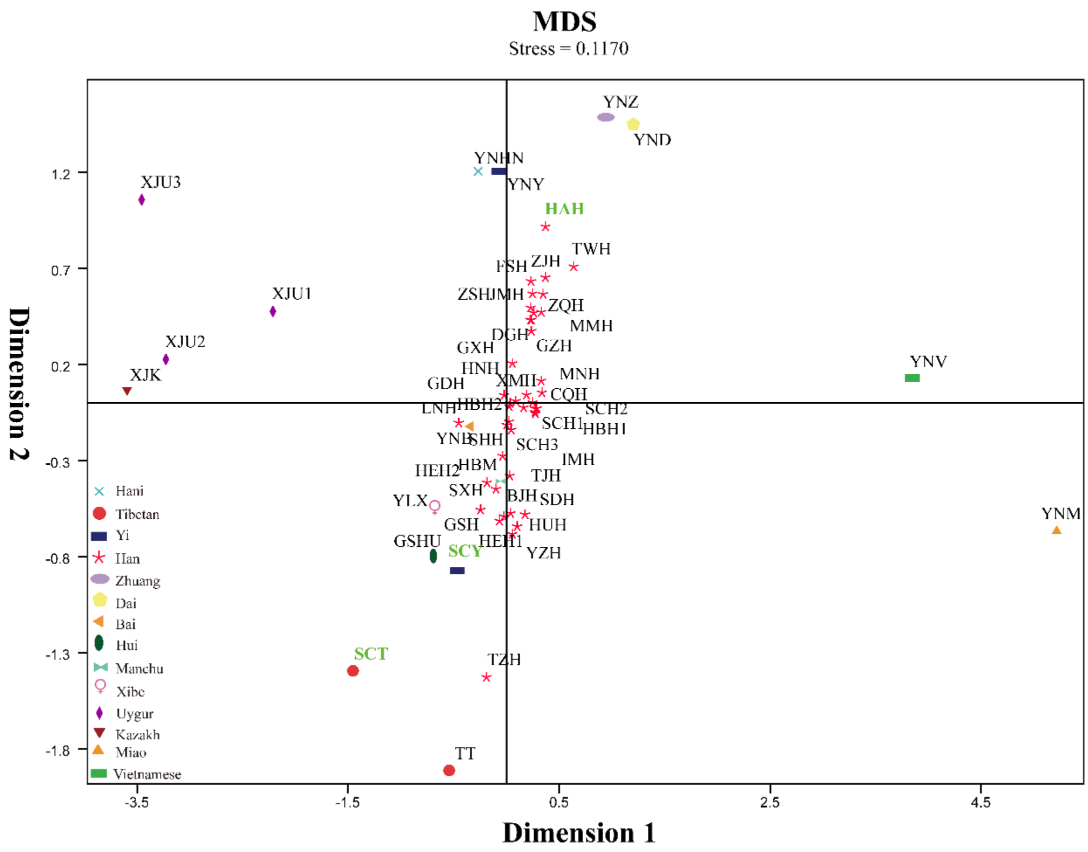

Supplementary Fig. S1. Multidimensional Scaling plots of our studied populations (bold and green) and 56 reference Chinese populations (the information of abbreviations is presented in **Table S12**).

Supplementary Table S1. p-values of Linkage disequilibrium test between all pairs of 23 STR loci in Hainan Han population（n=193）

Loci	[01]	[02]	[03]	[04]	[05]	[06]	[07]	[08]	[09]	[10]	[11]	[12]	[13]	[14]	[15]	[16]	[17]	[18]	[19]	[20]	[21]	[22]	[23]
[01]D3S1358																							
[02]vWA	0.9616																						
[03]D16S539	0.2496	0.0816																					
[04]CSF1PO	0.4561	0.7049	0.5201																				
[05]TPOX	0.6579	0.9152	0.8942	0.2030																			
[06]D8S1179	0.8285	0.4063	0.9051	0.1885	0.4525																		
[07]D21S11	0.6951	0.4327	0.9643	0.0166	0.5572	0.8417																	
[08]D18S51	0.8837	0.2084	0.3912	0.9697	0.5865	0.6551	0.0212																
[09]Penta E	0.4728	0.5986	0.1746	0.5303	0.4732	0.5355	0.4227	0.9799															
[10]D2S441	0.4017	0.8737	0.1356	0.0334	0.3720	0.2979	0.7025	0.7422	0.6789														
[11]D19S433	0.0961	0.6481	0.2276	0.1278	0.9288	0.7126	0.3991	0.5617	0.4901	0.5114													
[12]TH01	0.2438	0.5868	0.6328	0.8553	0.8476	0.9976	0.1356	0.2449	0.5794	0.1310	0.6471												
[13]FGA	0.4818	0.7710	0.0122	0.1765	0.7852	0.2857	0.2491	0.5625	0.2113	0.4204	0.3743	0.6444											
[14]D22S1045	0.7005	0.9809	0.0122	0.2838	0.0831	0.1696	0.9394	0.2020	0.3433	0.0535	0.8129	0.6697	0.0146										
[15]D5S818	0.8501	0.2158	0.4934	0.2025	0.1298	0.6560	0.2471	0.9398	0.0072	0.0841	0.5586	0.0171	0.4464	0.5829									
[16]D13S317	0.0273	0.0511	0.4607	0.8150	0.9434	0.6275	0.9103	0.6154	0.0562	0.4695	0.2285	0.2527	0.0620	0.1528	0.4995								
[17]D7S820	0.2815	0.5063	0.2905	0.5793	0.7337	0.9609	0.0457	0.7156	0.1755	0.0782	0.5122	0.6831	0.9891	0.4661	0.4143	0.5719							
[18]D6S1043	0.0959	0.0959	0.9415	0.4430	0.4467	0.0198	0.6724	0.7324	0.1605	0.2330	0.8113	0.2784	0.8508	0.4724	0.8788	0.6669	0.1221						
[19]D10S1248	0.7809	0.2652	0.7389	0.6502	0.1627	0.0606	0.9866	0.0731	0.8884	0.1152	0.5686	0.6595	0.3165	0.8287	0.2672	0.8992	0.7167	0.1654					
[20]D1S1656	0.1213	0.8208	0.5106	0.4252	0.0623	0.3540	0.3389	0.0919	0.0452	0.4273	0.2421	0.0871	0.0813	0.0034	0.7946	0.4096	0.5388	0.7389	0.9864				
[21]D12S391	0.2736	0.8524	0.6512	0.4625	0.3440	0.3122	0.4762	0.2983	0.9162	0.4200	0.2478	0.1545	0.1672	0.4717	0.7594	0.2870	0.5996	0.6072	0.1081	0.4118			
[22]D2S1338	0.5514	0.5290	0.5290	0.3720	0.0450	0.0855	0.2328	0.8119	0.5447	0.2655	0.5255	0.3306	0.1263	0.3034	0.3385	0.0151	0.7726	0.0653	0.6636	0.6280	0.4329		
[23]Penta D	0.9376	0.2544	0.7356	0.2461	0.6616	0.2433	0.0455	0.7637	0.3379	0.5817	0.9963	0.7516	0.9694	0.5701	0.8238	0.0888	0.3856	0.4775	0.4890	0.8898	0.6142	0.5009	

Supplementary Table S2. p-values of Linkage disequilibrium test between all pairs of 23 STR loci in Sichuan Yi population (n=177).

Loci	[01]	[02]	[03]	[04]	[05]	[06]	[07]	[08]	[09]	[10]	[11]	[12]	[13]	[14]	[15]	[16]	[17]	[18]	[19]	[20]	[21]	[22]	[23]
[01]D3S1358																							
[02]vWA	0.0093																						
[03]D16S539	0.8975	0.1048																					
[04]CSF1PO	0.2296	0.8761	0.3596																				
[05]TPOX	0.2991	0.8693	0.7783	0.6528																			
[06]D8S1179	0.0774	0.5353	0.7678	0.6493	0.5042																		
[07]D21S11	0.4426	0.0149	0.0267	0.2840	0.2258	0.6819																	
[08]D18S51	0.0043	0.1200	0.2522	0.6515	0.8391	0.7600	0.7692																
[09]Penta E	0.4040	0.3966	0.5052	0.9771	0.6545	0.3249	0.0578	0.6089															
[10]D2S441	0.0805	0.1348	0.8938	0.5898	0.8869	0.1939	0.3389	0.8820	0.7640														
[11]D19S433	0.5126	0.8259	0.8978	0.7831	0.6976	0.5715	0.0062	0.0885	0.8319	0.1102													
[12]TH01	0.2326	0.0926	0.4575	0.2721	0.9973	0.3047	0.8133	0.7343	0.6336	0.7241	0.0802												
[13]FGA	0.9273	0.7304	0.7660	0.9126	0.2162	0.1690	0.3315	0.4643	0.0816	0.0452	0.3750	0.0090											
[14]D22S1045	0.3988	0.6294	0.6733	0.8947	0.4327	0.4207	0.9866	0.5085	0.3987	0.1857	0.5415	0.3773	0.5496										
[15]D5S818	0.7827	0.6451	0.9049	0.7126	0.4445	0.6520	0.3996	0.7767	0.6318	0.1481	0.3355	0.5753	0.3817	0.8102									
[16]D13S317	0.0311	0.4655	0.0374	0.7346	0.6955	0.8549	0.6661	0.1278	0.9909	0.2740	0.1527	0.3018	0.9469	0.2248	0.7263								
[17]D7S820	0.7152	0.8378	0.7667	0.1487	0.5693	0.5745	0.1654	0.2600	0.0069	0.4546	0.2688	0.3647	0.9548	0.4314	0.7021	0.5668							
[18]D6S1043	0.5573	0.9137	0.2755	0.7645	0.8810	0.9037	0.4669	0.1406	0.1260	0.4066	0.6220	0.4033	0.2306	0.7683	0.2180	0.1966	0.4528						
[19]D10S1248	0.2658	0.0430	0.1291	0.0163	0.1761	0.3098	0.5546	0.6083	0.1376	0.0289	0.5324	0.0836	0.7937	0.9034	0.9225	0.9966	0.4161	0.7235					
[20]D1S1656	0.8179	0.1555	0.6394	0.8242	0.5238	0.1926	0.0022	0.2250	0.2850	0.3151	0.7146	0.5298	0.0697	0.3495	0.5861	0.1091	0.2682	0.5371	0.2393				
[21]D12S391	0.2619	0.8756	0.5339	0.0326	0.5199	0.5266	0.4253	0.2171	0.5198	0.3569	0.7395	0.0469	0.0979	0.2963	0.1518	0.9566	0.4863	0.2410	0.7055	0.2613			
[22]D2S1338	0.3532	0.1013	0.4022	0.8093	0.9505	0.7577	0.1847	0.3110	0.2624	0.6516	0.5655	0.1116	0.6388	0.2540	0.5068	0.2093	0.1616	0.2154	0.5896	0.1572	0.3474		
[23]Penta D	0.1756	0.2645	0.4687	0.3425	0.2451	0.8217	0.4625	0.3524	0.2171	0.8102	0.6254	0.9453	0.2136	0.3258	0.6795	0.3694	0.0527	0.2157	0.4761	0.0415	0.8883	0.1232	

Supplementary Table S3. p-values of Linkage disequilibrium test between all pairs of 23 STR loci in Sichuan Tibetan population (n=198).

Loci	[01]	[02]	[03]	[04]	[05]	[06]	[07]	[08]	[09]	[10]	[11]	[12]	[13]	[14]	[15]	[16]	[17]	[18]	[19]	[20]	[21]	[22]	[23]
[01]D3S1358																							
[02]vWA	0.2424																						
[03]D16S539	0.1632	0.4554																					
[04]CSF1PO	0.0970	0.0172	0.8740																				
[05]TPOX	0.5319	0.4833	0.9433	0.4968																			
[06]D8S1179	0.9532	0.9049	0.8763	0.6771	0.6675																		
[07]D21S11	0.0013	0.4255	0.4085	0.9891	0.1978	0.5154																	
[08]D18S51	0.9609	0.7474	0.0364	0.3890	0.2160	0.0084	0.0106																
[09]PentaE	0.1749	0.7938	0.9838	0.6826	0.4163	0.7230	0.0418	0.0661															
[10]D2S441	0.6280	0.4572	0.6512	0.5358	0.3334	0.0012	0.5363	0.3990	0.4155														
[11]D19S433	0.1441	0.7315	0.2584	0.2582	0.0522	0.7189	0.0891	0.5021	0.4618	0.8842													
[12]TH01	0.0706	0.3763	0.1170	0.6989	0.9828	0.2496	0.0107	0.1196	0.7198	0.3468	0.4144												
[13]FGA	0.3802	0.1957	0.2809	0.3854	0.8440	0.3697	0.7105	0.1098	0.0068	0.4876	0.0725	0.2462											
[14]D22S1045	0.1906	0.9643	0.3147	0.5924	0.9541	0.4073	0.7377	0.4216	0.5896	0.9678	0.4785	0.2869	0.1029										
[15]D5S818	0.2632	0.4251	0.5930	0.9831	0.7355	0.6059	0.9645	0.8784	0.8363	0.4456	0.5300	0.4203	0.4579	0.0015									
[16]D13S317	0.2385	0.0066	0.8466	0.2724	0.4810	0.7341	0.8713	0.3813	0.2192	0.2345	0.0452	0.6860	0.4052	0.7541	0.9296								
[17]D7S820	0.6004	0.3365	0.6133	0.1792	0.0625	0.2585	0.4410	0.6058	0.9838	0.1070	0.6078	0.4308	0.3391	0.7310	0.8375	0.8637							
[18]D6S1043	0.0686	0.5551	0.3895	0.3597	0.3824	0.3736	0.2404	0.0140	0.0421	0.4211	0.2557	0.7881	0.1664	0.5248	0.6218	0.5340	0.0936						
[19]D10S1248	0.5149	0.6168	0.0509	0.3834	0.7718	0.9779	0.2468	0.0273	0.0292	0.3702	0.5693	0.8675	0.9245	0.4324	0.1367	0.8116	0.6273	0.5287					
[20]D1S1656	0.1187	0.7127	0.1376	0.6538	0.0922	0.9969	0.5561	0.8315	0.0753	0.1026	0.5338	0.2028	0.1465	0.0178	0.1212	0.1590	0.5141	0.0545	0.7545				
[21]D12S391	0.3478	0.9239	0.0118	0.6167	0.9947	0.5374	0.4804	0.1267	0.3761	0.2506	0.3725	0.1123	0.3846	0.9031	0.2480	0.2803	0.7656	0.2567	0.2763	0.8651			
[22]D2S1338	0.0189	0.2648	0.6757	0.2937	0.9411	0.0021	0.9884	0.6101	0.1420	0.6539	0.2882	0.0278	0.2017	0.3485	0.2828	0.4097	0.4114	0.9474	0.1659	0.8841	0.3358		
[23]Penta D	0.2576	0.7941	0.3600	0.3266	0.1643	0.8475	0.3795	0.6553	0.9864	0.0068	0.4840	0.5377	0.6032	0.6506	0.7214	0.0504	0.1534	0.0059	0.0084	0.5641	0.0434	0.7512	

Supplementary Table S4. Allele frequencies for 23 autosomal STR loci of the Huaxia Platinum system in Hainan Han (n = 193).

Allele	D3S1358	vWA	D16S539	CSF1PO	TPOX	D8S1179	D21S11	D18S51	Penta E	D2S441	D19S433	TH01	FGA	D22S1045	D5S818	D13S317	D7S820	D6S1043	D10S1248	D1S1656	D12S391	D2S1338	Penta D
5									0.0466														
6												0.1010											0.0026
7				0.0130								0.2435			0.0311	0.0052	0.0052						0.0155
8			0.0078		0.5207				0.0026			0.0725				0.2694	0.1166						0.0544
9			0.2824	0.0363	0.1425				0.0155	0.0052		0.4793			0.0751	0.1503	0.0725	0.0181					0.4067
9.1										0.0389							0.0026						
9.3												0.0233											
9.4									0.0130														
10			0.1166	0.2461	0.0285	0.1062		0.0026	0.0440	0.2098		0.0725		0.0026	0.1995	0.1477	0.1762	0.0674					0.1166
10.3																		0.0052					
11			0.2694	0.2979	0.2902	0.0959		0.0026	0.1399	0.3808	0.0052	0.0052		0.1995	0.2824	0.2461	0.3705	0.0959	0.0026	0.0777			0.1166
11.2											0.0026												0.0026
11.3										0.0415													
12			0.2021	0.3238	0.0181	0.1528		0.0518	0.1140	0.1865	0.0492	0.0026		0.0026	0.2358	0.1347	0.2124	0.1839	0.0777	0.0440			0.1347
12.2											0.0026												
13	0.0026		0.1062	0.0751		0.1658		0.1788	0.0622	0.0104	0.2720		0.0078		0.1684	0.0363	0.0389	0.1295	0.3420	0.1088			0.0907
13.2											0.0337												
14	0.0363	0.3005	0.0155	0.0078		0.1762		0.2098	0.0699	0.1166	0.2176			0.0363	0.0078	0.0078	0.0052	0.0674	0.2461	0.1114			0.0466
14.1										0.0052													
14.2											0.1373												
15	0.3342	0.0181				0.2124		0.1917	0.0751	0.0052	0.0777			0.2746		0.0026		0.0078	0.2021	0.2876	0.0052		0.0104
15.2											0.1528												
16	0.3031	0.1477				0.0777		0.1269	0.0881		0.0078			0.2772				0.0337	0.1114	0.1762	0.0052	0.0207	
16.2											0.0337												
16.3																				0.0052			
17	0.2772	0.2254				0.0130		0.0829	0.0959		0.0052			0.1788				0.1399	0.0181	0.0984	0.0881	0.0803	
17.2											0.0026							0.0026					
17.3																				0.0570			
18	0.0440	0.2073						0.0570	0.0751				0.0207	0.0259				0.1891		0.0078	0.1917	0.1088	0.0026

18.3									0.0233		
18.4					0.0052						
19	0.0026	0.0829		0.0181	0.0440	0.0440	0.0026	0.0466	0.0026	0.1865	0.1995
19.3								0.0026			
20		0.0130		0.0337	0.0492	0.0544		0.0078		0.1917	0.1114
20.3								0.0026			
21		0.0052		0.0130	0.0259	0.1528				0.1399	0.0363
21.2						0.0052					
22				0.0181	0.0130	0.1917				0.0984	0.0492
22.2						0.0078					
23				0.0104	0.0130	0.2047				0.0570	0.1425
23.2						0.0104					
24				0.0026	0.0078	0.1192				0.0181	0.1788
24.2						0.0130					
25						0.0933				0.0130	0.0648
25.2						0.0104					
26						0.0466				0.0026	0.0078
26.2						0.0026					
27			0.0052			0.0155				0.0026	
28			0.0544								
28.2			0.0052								
29			0.2746								
30			0.2539								
30.2			0.0104								
30.3			0.0026								
31			0.0907								
31.2			0.0622								
32			0.0415								
32.2			0.1295								
33			0.0078								
33.1			0.0026								

33.2	0.0570
34.2	0.0026

Supplementary Table S5. Allele frequencies for 23 autosomal STR loci of the Huaxia Platinum system in Sichuan Yi (n = 177).

Allele	D3S1358	vWA	D16S539	CSF1PO	TPOX	D8S1179	D21S11	D18S51	Penta E	D2S441	D19S433	TH01	FGA	D22S1045	D5S818	D13S317	D7S820	D6S1043	D10S1248	D1S1656	D12S391	D2S1338	Penta D
5									0.0198														
6												0.0819				0.0028							0.0056
7				0.0113					0.0028			0.2994			0.0198								0.0113
8			0.0367		0.5056	0.0028			0.0085			0.0424			0.0028	0.2655	0.1667	0.0085					0.0367
8.4									0.0056														
9			0.2203	0.0226	0.1271				0.0226	0.0028		0.4859			0.0593	0.1045	0.0819	0.0028					0.3503
9.1										0.0113													
9.3												0.0508											
10	0.0028		0.0960	0.1921	0.0113	0.0989			0.0028	0.2599		0.0395			0.1977	0.1667	0.1638	0.0311					0.1102
10.1																	0.0141						
10.2											0.0028												
11			0.2571	0.2458	0.3079	0.0424			0.1469	0.3023				0.3107	0.3079	0.2260	0.2571	0.1328	0.0085	0.0763			0.1554
11.3										0.0367													
12			0.2627	0.4435	0.0480	0.1412		0.0141	0.1073	0.1441	0.0311			0.0028	0.2373	0.1638	0.2627	0.1158	0.0678	0.0141			0.1271
12.1						0.0028																	
12.2											0.0028												
12.3						0.0028																	
13			0.0989	0.0819		0.2373		0.2458	0.0367	0.0367	0.2938				0.1667	0.0565	0.0480	0.1497	0.3729	0.1271			0.1667
13.2										0.0028	0.0367												
14	0.0311	0.1977	0.0226	0.0028		0.1949		0.2345	0.1158	0.1412	0.2232		0.0028	0.0169	0.0085	0.0141	0.0056	0.1271	0.2373	0.1102	0.0028		0.0226
14.1										0.0424													
14.2											0.1186												
15		0.0226	0.0056			0.1921		0.1158	0.1045	0.0169	0.0593			0.2090				0.0169	0.1751	0.2571	0.0028		0.0141
15.2											0.1356												
16	0.3701	0.2571				0.0791		0.0847	0.0876	0.0028	0.0282			0.2316				0.0056	0.1073	0.2542	0.0028	0.0141	
16.2											0.0621												
17	0.1638	0.2429				0.0056		0.0989	0.0508		0.0028			0.2062				0.0226	0.0311	0.1102	0.1158	0.0621	
17.2											0.0028												
17.3																				0.0424			

18	0.0508	0.1610		0.0339	0.1130	0.0311	0.0198	0.1695	0.0028	0.2175	0.1158
18.2								0.0056			
18.3									0.0028	0.0028	
19		0.1017		0.0565	0.0650	0.0537	0.0028	0.1328		0.2175	0.1949
19.3								0.0028	0.0028		
20		0.0169		0.0424	0.0593	0.0763		0.0537		0.1667	0.1243
20.3								0.0056			
21				0.0311	0.0198	0.0678		0.0028		0.1017	0.0141
21.1				0.0056							
21.2						0.0056					
21.3								0.0085			
22				0.0226	0.0198	0.1525				0.0791	0.0282
22.2						0.0028					
22.3								0.0056			
23				0.0028	0.0085	0.2684				0.0565	0.2288
23.2						0.0056					
24				0.0056		0.1667				0.0254	0.1356
24.2						0.0056					
25				0.0028		0.0876				0.0056	0.0706
26					0.0028	0.0508				0.0028	0.0056
27						0.0141					0.0056
28			0.0452			0.0085					
28.2			0.0254								
29			0.2401								
29.2			0.0028								
30			0.2655	0.0028							
30.2			0.0395								
31			0.0932								
31.2			0.0734								
32			0.0367								
32.2			0.1102								

32.3	0.0028
33	0.0028
33.2	0.0537
34.2	0.0085

Supplementary Table S6. Allele frequencies for 23 autosomal STR loci of the Huaxia Platinum system in Sichuan Tibetan (n = 198).

Allele	D3S1358	vWA	D16S539	CSF1PO	TPOX	D8S1179	D21S11	D18S51	Penta E	D2S441	D19S433	TH01	FGA	D22S1045	D5S818	D13S317	D7S820	D6S1043	D10S1248	D1S1656	D12S391	D2S1338	Penta D
5									0.0278							0.0051							
6												0.0732											0.0025
7				0.0051								0.2652					0.0025						0.0051
8			0.0051		0.5808				0.0025			0.0783			0.0126	0.2551	0.1768	0.0025					0.0833
9			0.1843	0.0404	0.1818				0.0101	0.0025		0.5076			0.0808	0.0909	0.0505						0.2854
9.1										0.0126							0.0025						
9.3												0.0606											
10			0.1667	0.2652	0.0101	0.0530			0.0328	0.2727		0.0126			0.1768	0.1162	0.2071	0.0152					0.1338
10.2										0.0025													
11			0.3990	0.2803	0.2071	0.0429			0.1237	0.3510				0.3106	0.3157	0.2475	0.2399	0.1288	0.0025	0.0328			0.2551
11.3										0.0152													
12			0.1616	0.3308	0.0177	0.2020		0.0076	0.1111	0.1869	0.0177	0.0025			0.2626	0.1919	0.2702	0.0934	0.0631	0.0808			0.1061
12.2											0.0076												
13			0.0783	0.0581	0.0025	0.2677		0.3207	0.0253	0.0429	0.2399				0.1439	0.0657	0.0429	0.1591	0.3712	0.1263			0.1061
13.2											0.0404												
14	0.0303	0.2273	0.0051	0.0202		0.1894		0.2071	0.0783	0.1035	0.2399			0.0177	0.0051	0.0278	0.0076	0.1414	0.2399	0.0631			0.0152
14.1										0.0025													
14.2											0.1591												
15	0.3535	0.0101				0.1540		0.1742	0.1086	0.0051	0.0985			0.1768	0.0025			0.0051	0.1970	0.2222	0.0152		0.0076
15.2											0.1136												
16	0.3990	0.1944				0.0859		0.1010	0.0985	0.0025	0.0278			0.2753					0.0960	0.3333	0.0126	0.0025	
16.2											0.0429												
16.3																				0.0025			
17	0.1616	0.2727				0.0051		0.0682	0.0657		0.0076			0.2146				0.0379	0.0303	0.0934	0.1061	0.0278	
17.2											0.0051												
17.3																		0.0051		0.0253			
18	0.0530	0.1540						0.0177	0.0808				0.0126	0.0051				0.1692		0.0101	0.2576	0.0758	
18.3																				0.0076			
19	0.0025	0.1313						0.0278	0.0783				0.0505					0.1364		0.0025	0.2222	0.2121	

19.3						0.0101		
20	0.0076		0.0303	0.0556	0.0328	0.0783	0.1667	0.1111
20.3						0.0051		
21	0.0025		0.0202	0.0505	0.0707		0.0682	0.0455
21.3						0.0126		
22			0.0177	0.0253	0.1742		0.0783	0.0379
22.2					0.0051			
23			0.0025	0.0227	0.2020		0.0657	0.2525
23.2					0.0278			
24			0.0025		0.2096		0.0051	0.1566
24.2					0.0101			
25			0.0025		0.1263		0.0025	0.0556
25.2					0.0025			
26				0.0025	0.0379			0.0202
26.2					0.0076			
27					0.0177			0.0025
28		0.0455			0.0126			
28.2		0.0177						
29		0.2576						
29.2		0.0051						
30		0.2702						
30.2		0.0126						
31		0.0732						
31.2		0.1035						
32		0.0404						
32.2		0.0934						
33.2		0.0707						
34.2		0.0101						

Supplementary Table S7. Forensic parameters of the Huaxia Platinum System and three compared multiplex amplication kits in three investigated populations.

Multiplex Amplication Kit	Hainan Han		Sichuan Yi		Sichuan Tibetan	
	CPD	CPE	CPD	CPE	CPD	CPE
Huaxia Platinum System	0.9999999999999999999999999992	0.9999999999	0.999999999999999999999999999	0.999999998	0.999999999999999999999999992	0.999999995
PowerPlex 21 Kit	0.999999999999999999999999	0.999999999	0.999999999999999999999997	0.99999998	0.999999999999999999999993	0.99999996
Goldeneye DNA ID System 20A	0.99999999999999999999998	0.999999996	0.99999999999999999999996	0.99999996	0.9999999999999999999999	0.99999991
AGCU EX22 Kit	0.9999999999999999999999998	0.9999999991	0.9999999999999999999999997	0.999999991	0.999999999999999999999999	0.99999998

CPD: combined power of discrimination; CPE: combined power of exclusion.

Supplementary Table S8. The Fst and corresponding p value of locus-by-locus pairwise comparisons between the Hainan Han and other 58 relative reference populations.

Loci		Tibet-Tibetan	Sichuan-Han1	Sichuan-Han2	Sichuan-Han3	Chongqing-Han	GansuPingliang-Han	InnerMongolia-Han	Hunan-Han	Henan-Han1	Henan-Han2	Hubei-Han1	Hubei-Han2	Jiangxi-Han	Guangxi-Han	Guangdong-Han
CSF1PO	Fst	0.0047	0.0029	0.0046	0.0013	0.0021	-0.0006	0.0009	0.0079	0.0022	-0.0004	0.0010	0.0018	0.0020	0.0026	0.0036
	p	0.1771	0.1259	0.0431	0.2522	0.1661	0.4426	0.2826	0.0154	0.1628	0.4166	0.2592	0.1668	0.1779	0.1278	0.0760
D2S1338	Fst	0.0022	0.0002	0.0016	0.0014	0.0013	-0.0005	0.0034	0.0004	0.0033	0.0023	0.0009	0.0007	-0.0011	-0.0016	-0.0003
	p	0.2362	0.3773	0.1339	0.2178	0.2021	0.4835	0.0668	0.3334	0.0570	0.1446	0.2626	0.2505	0.6900	0.8555	0.4889
D3S1358	Fst	-0.0040	-0.0001	-0.0007	0.0013	-0.0006	0.0008	0.0033	0.0006	0.0047	0.0007	-0.0003	0.0012	-0.0004	-0.0019	0.0009
	p	0.7045	0.3809	0.5081	0.2615	0.4730	0.3165	0.1292	0.3058	0.0715	0.3132	0.4246	0.2176	0.4335	0.7473	0.2589
D5S818	Fst	0.0033	-0.0012	-0.0010	-0.0031	-0.0025	-0.0035	0.1131	-0.0028	-0.0005	0.0012	-0.0002	-0.0005	-0.0007	-0.0020	-0.0016
	p	0.2104	0.6172	0.6363	0.9177	0.9204	0.9046	0.0000	0.9758	0.4762	0.2685	0.4263	0.5030	0.5310	0.8374	0.7718
D6S1043	Fst	0.0023	0.0123	0.0155	0.0124	0.0180	0.0232	0.0159	0.0218	0.0340	0.0194	0.0161	0.0164	0.0191	0.0161	0.0159
	p	0.2200	0.0000	0.0000	0.0001	0.0000	0.0000	0.0000	0.0000	0.0000	0.0000	0.0000	0.0000	0.0000	0.0000	0.0000
D7S820	Fst	0.0074	-0.0006	-0.0017	-0.0022	-0.0009	-0.0015	-0.0029	-0.0006	-0.0006	-0.0022	-0.0010	-0.0013	-0.0019	-0.0013	-0.0016
	p	0.0861	0.4918	0.8206	0.7678	0.5594	0.6031	0.9367	0.5091	0.4971	0.7307	0.5838	0.7264	0.7844	0.6674	0.7732
D8S1179	Fst	0.0016	-0.0007	0.00007	-0.0009	0.0014	0.0036	-0.0017	0.0021	0.0025	-0.0007	0.0008	0.0007	0.0004	0.0019	-0.0011
	p	0.2868	0.5772	0.3949	0.5780	0.1962	0.1107	0.7643	0.1275	0.1164	0.5293	0.2890	0.2721	0.3438	0.1465	0.7016
D12S391	Fst	0.0005	-0.0018	-0.0013	-0.0007	-0.0007	-0.0014	-0.0006	-0.0017	0.0025	0.0003	0.0001	-0.0008	-0.0022	-0.0019	-0.0015
	p	0.3797	0.8587	0.7833	0.5415	0.5536	0.6316	0.5409	0.8274	0.0985	0.3697	0.3904	0.6382	0.9218	0.8829	0.8286
D13S317	Fst	0.0024	-0.0015	-0.0018	-0.0025	-0.0022	-0.0007	-0.0022	-0.0028	-0.0024	-0.0017	-0.0019	-0.0018	-0.0022	-0.0007	-0.0022
	p	0.2405	0.7029	0.8661	0.8386	0.8779	0.5025	0.8182	0.9815	0.8959	0.6709	0.7999	0.8666	0.8584	0.5356	0.9302
D16S539	Fst	-0.0016	-0.0033	-0.0026	-0.0011	-0.0025	-0.0041	0.0825	-0.0025	-0.0028	-0.0040	-0.0023	-0.0018	-0.0016	0.0005	-0.0020
	p	0.5236	0.9993	0.9931	0.5558	0.9133	0.9757	0.0000	0.9227	0.9466	0.9855	0.8532	0.8443	0.7157	0.3103	0.8819
D18S51	Fst	0.0052	-0.0022	-0.0018	-0.0028	-0.0023	-0.0029	-0.0023	-0.0024	-0.0017	-0.0030	-0.0020	-0.0014	-0.0022	0.0003	-0.0018
	p	0.1001	0.9542	0.9563	0.9538	0.9620	0.9211	0.9222	0.9826	0.8493	0.9610	0.9092	0.8603	0.9383	0.3477	0.9296
D19S433	Fst	-0.0053	-0.0027	-0.0020	-0.0036	-0.0026	-0.0023	-0.0018	-0.0024	-0.0028	-0.0000 3	-0.0026	-0.0016	-0.0022	-0.0021	-0.0009
	p	0.9560	0.9852	0.9595	0.9953	0.9792	0.7841	0.7864	0.9567	0.9904	0.3972	0.9668	0.8656	0.9077	0.9135	0.6339
D21S11	Fst	-0.0003	-0.0022	-0.0022	-0.0031	-0.0027	-0.0033	-0.0022	-0.0013	-0.0015	-0.0031	-0.0021	-0.0010	-0.0025	-0.0013	-0.0013
	p	0.4315	0.9307	0.9843	0.9762	0.9890	0.9406	0.8803	0.7102	0.7574	0.9579	0.8893	0.6864	0.9557	0.7264	0.7708
FGA	Fst	0.0070	-0.0015	0.0004	0.0037	0.0016	0.0022	0.0225	-0.0005	0.0056	0.0064	0.0021	0.0011	0.0019	-0.00009	0.0006
	p	0.0491	0.8176	0.3231	0.0696	0.1639	0.1755	0.0000	0.5424	0.0143	0.0241	0.1294	0.1936	0.1397	0.4473	0.2830

PentaD	Fst	0.0168	0.0022	0.0022	0.0000	0.0017	0.0093	0.0040	0.0008	0.0083	0.0062	0.0045	0.0016	0.0021	-0.0016	0.0028
	p	0.0077	0.1294	0.1018	0.3917	0.1738	0.0158	0.0689	0.2715	0.0060	0.0357	0.0438	0.1532	0.1505	0.7753	0.0748
PentaE	Fst	-0.0039	-0.0008	-0.0009	-0.0026	-0.0013	0.0006	0.0163	-0.0016	-0.0013	-0.0031	-0.0017	-0.0015	-0.0012	0.0025	-0.0014
	p	0.9449	0.6971	0.7862	0.9772	0.8325	0.3263	0.0000	0.9233	0.8569	0.9930	0.9173	0.9559	0.8031	0.0597	0.9280
TH01	Fst	0.0067	-0.0015	-0.0003	0.0002	-0.0011	0.0032	0.0005	0.0003	-0.0003	0.0006	0.0010	-0.0006	-0.0016	0.0022	-0.0002
	p	0.1178	0.6551	0.4116	0.3472	0.5648	0.1637	0.3101	0.3230	0.4136	0.3047	0.2458	0.4708	0.6556	0.1553	0.3989
TPOX	Fst	-0.0001	-0.0027	-0.0023	-0.0039	-0.0028	-0.0024	-0.0023	-0.0026	-0.0018	-0.0009	-0.0027	-0.0023	-0.0024	-0.0008	-0.0026
	p	0.3663	0.8678	0.8902	0.9644	0.8945	0.6185	0.7209	0.8654	0.6461	0.4474	0.8463	0.9141	0.7770	0.4577	0.9407
VWA	Fst	0.0039	-0.0022	-0.0015	0.0017	-0.0020	0.0000	-0.0005	-0.0018	-0.0001	0.0024	-0.0002	-0.0003	-0.0014	-0.0030	-0.0008
	p	0.1811	0.8732	0.7889	0.2280	0.8159	0.3950	0.4775	0.7690	0.4098	0.1807	0.4327	0.4727	0.6783	0.9958	0.5797

Continue Table S8:

Loci		Guangzhou-Han	Dongguan-Han	Foshan-Han	Jiangmen-Han-	Maoming-Han	Qingyuan-Han	Yangjiang-Han	Zhanjiang-Han	Zhaoqing-Han	Zhongshan-Han	Minnan-Han	Minxi-Han	Xiamen-Han	Beijing-Han	Tianjin-Han
CSF1PO	Fst	0.0013	-0.0019	0.0027	0.0018	0.0012	0.0006	-0.0004	-0.0021	0.0034	0.0014	-0.0017	0.0026	0.0012	0.0065	-0.0018
	p	0.2095	0.7094	0.1220	0.1675	0.2482	0.3010	0.4340	0.7701	0.0896	0.2257	0.6538	0.1387	0.2108	0.0730	0.6005
D2S1338	Fst	-0.0008	-0.0010	-0.0010	-0.0023	-0.0029	-0.0014	-0.0018	-0.0024	-0.0014	-0.0008	0.0037	-0.0022	0.0006	0.0047	0.0001
	p	0.6739	0.6439	0.6873	0.9835	0.9833	0.8223	0.9256	0.9460	0.8063	0.6096	0.0620	0.9319	0.2788	0.0615	0.3986
D3S1358	Fst	-0.0014	-0.0031	0.0003	-0.0025	-0.0030	-0.0023	-0.0019	-0.0020	-0.0015	-0.0026	-0.0005	-0.0010	0.0003	-0.0009	0.0004
	p	0.6954	0.9636	0.3300	0.9520	0.9221	0.8872	0.8211	0.7289	0.6757	0.9039	0.4427	0.5217	0.3353	0.4731	0.3522
D5S818	Fst	-0.0018	-0.0019	-0.0019	-0.0025	0.0005	-0.0017	-0.0017	-0.0012	-0.0012	-0.0020	-0.0038	-0.0018	-0.0004	-0.0038	-0.0018
	p	0.8781	0.7491	0.8102	0.9668	0.3125	0.8016	0.8125	0.6201	0.6672	0.8070	0.9986	0.7635	0.4780	0.9156	0.6239
D6S1043	Fst	0.0170	0.0191	0.0175	0.0148	0.0162	0.0186	0.0161	0.0148	0.0171	0.0183	0.0197	0.0144	0.0167	0.0202	0.0144
	p	0.0000	0.0000	0.0000	0.0000	0.0000	0.0000	0.0000	0.0000	0.0000	0.0000	0.0000	0.0000	0.0000	0.0000	0.0000
D7S820	Fst	-0.0018	-0.0031	-0.0027	-0.0026	-0.0034	-0.0006	-0.0020	-0.0026	-0.0024	-0.0018	-0.0023	-0.0013	-0.0020	-0.0040	-0.0031
	p	0.8795	0.9796	0.9877	0.9769	0.9955	0.5174	0.8824	0.8984	0.9546	0.7615	0.7927	0.6382	0.9159	0.9444	0.8300
D8S1179	Fst	0.0005	-0.0008	0.0012	0.0005	0.0030	0.0027	0.0009	0.0007	0.0016	0.0015	-0.0006	-0.0006	-0.0004	0.0027	0.0002
	p	0.3106	0.5936	0.2085	0.3142	0.0938	0.0770	0.2397	0.2949	0.1566	0.1882	0.5281	0.5487	0.5397	0.1616	0.3922
D12S391	Fst	-0.0018	-0.0021	-0.0024	-0.0025	-0.0020	-0.0025	-0.0020	-0.0023	-0.0019	-0.0027	-0.0030	-0.0021	-0.0012	-0.0007	0.0005
	p	0.9335	0.8840	0.9771	0.9927	0.8376	0.9915	0.9597	0.8998	0.9265	0.9900	0.9736	0.9009	0.7815	0.5135	0.3475
D13S317	Fst	-0.0004	-0.0024	-0.0013	-0.0001	-0.0004	-0.0007	-0.0000 5	-0.0014	-0.0014	-0.0003	-0.0026	-0.0014	-0.0018	-0.0038	-0.0033
	p	0.4952	0.8764	0.6761	0.4070	0.4649	0.5407	0.4196	0.6576	0.7175	0.4606	0.8755	0.6787	0.8848	0.9332	0.8745

D16S539	Fst	-0.0008	-0.0025	0.0016	0.0003	-0.0026	-0.0011	-0.0007	-0.0014	-0.0013	0.0007	-0.0032	-0.0028	-0.0023	-0.0050	-0.0009
	p	0.5687	0.8800	0.1853	0.3552	0.8891	0.6376	0.5331	0.6282	0.7001	0.3006	0.9454	0.9510	0.9694	0.9999	0.5023
D18S51	Fst	-0.0020	-0.0026	-0.0015	-0.0015	-0.0011	-0.0020	-0.0013	-0.0014	-0.0010	-0.0010	-0.0025	-0.0024	-0.0020	-0.0003	-0.0036
	p	0.9772	0.9758	0.8411	0.8339	0.6725	0.9579	0.8197	0.7468	0.6893	0.6858	0.9168	0.9645	0.9845	0.4599	0.9695
D19S433	Fst	-0.0017	-0.0021	-0.0015	-0.0021	-0.0019	-0.0013	-0.0006	-0.0023	-0.0014	-0.0019	-0.0011	-0.0021	-0.0018	0.0000 2	-0.0034
	p	0.9029	0.8611	0.7789	0.9511	0.8086	0.7441	0.5328	0.8935	0.7745	0.8555	0.6015	0.8955	0.9243	0.3971	0.9271
D21S11	Fst	-0.0013	-0.0021	-0.0006	-0.0008	-0.0024	-0.0015	-0.0015	-0.0021	-0.0012	-0.0012	-0.0031	-0.0018	-0.0018	-0.0024	-0.0035
	p	0.8017	0.8588	0.5320	0.6095	0.9047	0.8085	0.8126	0.8642	0.7047	0.6881	0.9787	0.8232	0.9408	0.8007	0.9520
FGA	Fst	-0.0014	0.0010	-0.0012	-0.0005	-0.0015	-0.0010	-0.0015	-0.0013	-0.0008	-0.0013	-0.0006	-0.0004	0.0004	0.0020	-0.0009
	p	0.8546	0.2426	0.7588	0.5685	0.7722	0.6897	0.8738	0.7258	0.6472	0.7469	0.5329	0.5071	0.3005	0.1951	0.5530
PentaD	Fst	-0.0017	-0.0013	-0.0020	-0.0012	-0.0015	-0.0020	-0.0008	-0.0030	-0.0016	-0.0023	0.0011	-0.0009	0.0006	0.0066	0.0084
	p	0.8824	0.6612	0.8888	0.7052	0.7039	0.9044	0.5941	0.9847	0.8188	0.9369	0.2594	0.5984	0.2814	0.0424	0.0212
PentaE	Fst	0.0001	0.0004	0.0003	-0.0006	0.0006	-0.0005	0.0000 7	0.0015	0.0008	0.0002	-0.0021	-0.0009	-0.0013	-0.0032	-0.0037
	p	0.3872	0.3473	0.3422	0.6545	0.3122	0.5805	0.4066	0.1532	0.2234	0.3783	0.9400	0.7152	0.9080	0.9826	0.9972
TH01	Fst	0.0000	-0.0004	0.0036	0.0003	-0.0006	0.0011	0.0012	0.0000	0.0024	-0.0009	0.0017	-0.0020	-0.0013	-0.0007	-0.0007
	p	0.3731	0.4153	0.0819	0.3246	0.4618	0.2377	0.2246	0.3821	0.1341	0.5173	0.2204	0.7667	0.6872	0.4349	0.4387
TPOX	Fst	-0.0014	-0.0011	-0.0016	-0.0011	-0.0012	-0.0014	-0.0013	-0.0010	-0.0006	-0.0013	-0.0026	-0.0022	-0.0020	-0.0049	-0.0048
	p	0.6492	0.4827	0.6275	0.5365	0.4988	0.5990	0.5866	0.4780	0.4479	0.5430	0.7298	0.7380	0.8120	0.9933	0.9757
VWA	Fst	-0.0021	-0.0032	-0.0025	-0.0021	-0.0031	-0.0024	-0.0025	-0.0035	-0.0027	-0.0027	0.0009	-0.0008	-0.0004	0.0015	-0.0030
	p	0.9362	0.9898	0.9504	0.9018	0.9581	0.9522	0.9840	0.9989	0.9921	0.9616	0.2961	0.5554	0.4806	0.2598	0.8199

Continue Table S8:

Loci		Shanghai-Han	Jiangsu-Huai'an-Han	Jiangsu-Yangzhou-Han	Jiangsu-Taizhou-Han	Anhui-Suzhou-Han	Shangdong-Han	Shanxi-Han	Jiangxi-Jiujiang-Han	Yunnan-Han	Yungui-Han	Taiwan-Han	Xinjiang-Uyghur1	Xinjiang-Uyghur2	Xinjiang-Uyghur3	Xinjiang-Kazakh
CSF1PO	Fst	0.0031	0.0101	-0.0021	0.0054	0.0009	-0.0023	-0.0011	0.0021	0.0004	0.0021	0.0004	0.0007	-0.0035	0.0007	-0.0065
	p	0.1055	0.0428	0.6124	0.1371	0.2995	0.6690	0.5604	0.1357	0.3223	0.1455	0.3155	0.2783	0.8537	0.3306	0.9046
D2S1338	Fst	0.0008	0.0041	0.0007	-0.0009	-0.0004	0.0014	0.0023	0.0005	0.0005	0.0010	0.0013	0.0031	0.0019	0.0005	-0.0002
	p	0.2588	0.1142	0.3253	0.5224	0.4799	0.2508	0.1116	0.3112	0.3028	0.2163	0.1641	0.0478	0.2105	0.3751	0.4311
D3S1358	Fst	0.0004	0.0029	0.0038	-0.0045	0.0037	0.0009	-0.0001	0.0006	0.0007	-0.0008	-.0002	0.0015	0.0017	0.0043	0.0061
	p	0.3220	0.2120	0.1601	0.7957	0.1533	0.3007	0.3954	0.2950	0.2856	0.5355	0.4043	0.1936	0.2530	0.1895	0.1603
D5S818	Fst	-0.0017	-0.0047	-0.0028	-0.0017	-0.0029	-0.0042	-0.0007	-0.0019	-0.0015	-0.0015	-0.0021	0.0078	0.0096	0.0022	-0.0048
	p	0.7530	0.9212	0.7522	0.5452	0.7953	0.9678	0.5243	0.9069	0.7535	0.7559	0.9200	0.0095	0.0243	0.2611	0.7718

D6S1043	Fst	0.0162	0.0178	0.0117	0.0166	0.0148	0.0095	0.0127	0.0167	0.0161	0.0164	0.0151	0.0267	0.0214	0.0147	0.0341
	p	0.0000	0.0005	0.0018	0.0015	0.0001	0.0044	0.0001	0.0000	0.0000	0.0000	0.0000	0.0000	0.0000	0.0026	0.0000
D7S820	Fst	-0.0007	-0.0061	0.0009	-0.0017	-0.0045	-0.0037	-0.0015	-0.0019	-0.0021	-0.0015	-0.0023	0.0081	0.0075	0.0781	0.0140
	p	0.5270	0.9997	0.3162	0.5405	0.9844	0.9227	0.6959	0.9024	0.9206	0.7798	0.9582	0.0057	0.0369	0.0000	0.0327
D8S1179	Fst	0.0025	-0.0027	-0.0035	-0.0026	0.0009	-0.0010	0.0047	-0.0005	0.0000	0.0010	-0.0013	0.0084	0.0072	0.0000	0.0040
	p	0.1072	0.7365	0.9142	0.7075	0.3159	0.5592	0.0292	0.5626	0.4233	0.2233	0.7740	0.0021	0.0261	0.4078	0.1863
D12S391	Fst	-0.0012	-0.0025	-0.0012	-0.0029	0.0002	0.0018	0.0075	-0.0010	-0.0008	0.0004	-0.0004	0.0004	-0.0031	-0.0033	0.0078
	p	0.6878	0.7366	0.6016	0.7419	0.3742	0.2173	0.0067	0.7293	0.6097	0.3303	0.5241	0.3221	0.9171	0.7940	0.0794
D13S317	Fst	-0.0023	-0.0039	-0.0038	-0.0042	-0.0022	-0.0035	-0.0007	-0.0022	-0.0022	-0.0014	-0.0018	0.0142	0.0261	0.0018	0.0062
	p	0.9026	0.8398	0.9237	0.8457	0.6967	0.9010	0.5337	0.9728	0.9549	0.7683	0.8572	0.0000	0.0000	0.2847	0.1267
D16S539	Fst	-0.0013	-0.0036	-0.0027	-0.0030	-0.0021	-0.0034	-0.0035	-0.0020	-0.0020	-0.0022	-0.0024	0.0034	0.0015	0.0012	0.0011
	p	0.6503	0.7993	0.7417	0.6730	0.6737	0.8683	0.9999	0.9068	0.9090	0.9462	0.9746	0.0654	0.2484	0.3169	0.3433
D18S51	Fst	-0.0018	-0.0033	-0.0022	-0.0023	-0.0016	-0.0006	-0.0020	-0.0017	-0.0018	-0.0017	-0.0010	-0.0015	-0.0003	-0.0017	-0.0041
	p	0.8847	0.8481	0.7706	0.6922	0.6689	0.5075	0.9024	0.9282	0.9379	0.9194	0.7169	0.8463	0.4653	0.6057	0.8069
D19S433	Fst	-0.0021	-0.0036	-0.0032	-0.0065	-0.0016	-0.0024	-0.0014	-0.0017	-0.0016	-0.0021	-0.0022	0.0045	0.0082	0.0010	0.0144
	p	0.8952	0.8528	0.8838	0.9996	0.6460	0.7834	0.7018	0.9141	0.8576	0.9777	0.9708	0.0243	0.0210	0.3210	0.0200
D21S11	Fst	-0.0020	0.0004	-0.0026	-0.0034	0.0029	-0.0025	-0.0011	-0.0016	-0.0011	-0.0018	-0.0016	0.0013	0.0036	-0.0025	0.0007
	p	0.8922	0.3564	0.7922	0.7965	0.1398	0.8052	0.6629	0.8998	0.7027	0.9284	0.8602	0.1809	0.1120	0.6769	0.3417
FGA	Fst	0.0007	-0.0025	0.0000	-0.0010	0.0044	0.0080	0.0025	0.0007	0.0010	0.0012	-0.0004	0.0043	0.0005	0.0002	-0.0021
	p	0.2860	0.7488	0.4048	0.5163	0.0637	0.0154	0.1002	0.2572	0.2120	0.1809	0.5351	0.0201	0.3515	0.3820	0.6144
PentaD	Fst	0.0038	0.0088	0.0160	0.0036	0.0058	0.0049	0.0040	0.0027	0.0022	0.0036	0.0012	0.0119	0.0257	0.0032	0.0210
	p	0.0552	0.0362	0.0020	0.1769	0.0582	0.0823	0.0552	0.0747	0.1037	0.0386	0.1927	0.0003	0.0000	0.1943	0.0050
PentaE	Fst	-0.0012	0.0005	-0.0032	-0.0004	-0.0024	-0.0025	-0.0017	-0.0014	-0.0015	-0.0011	-0.0008	0.0008	0.0013	0.0005	-0.0012
	p	0.8074	0.3718	0.9679	0.4904	0.9137	0.9251	0.9275	0.9274	0.9433	0.8253	0.7195	0.2248	0.2330	0.3835	0.6024
TH01	Fst	-0.0010	-0.0027	0.0018	0.0073	-0.0002	0.0021	0.0010	-0.0006	0.0000	0.0005	0.0001	0.0333	0.0458	0.0314	0.0381
	p	0.5341	0.6414	0.2293	0.1027	0.3990	0.2180	0.2499	0.4865	0.3666	0.2969	0.3538	0.0000	0.0000	0.0012	0.0008
TPOX	Fst	-0.0025	-0.0039	-0.0038	-0.0061	-0.0026	-0.0042	-0.0020	-0.0019	-0.0019	-0.0012	-0.0001	0.0009	-0.0017	-0.0022	0.0036
	p	0.8382	0.7174	0.7848	0.9411	0.6283	0.8938	0.6795	0.7785	0.7595	0.5562	0.3693	0.2491	0.5295	0.5081	0.2337
VWA	Fst	0.0026	0.0022	-0.0002	-0.0054	0.0007	0.0004	0.0003	-0.0005	-0.0004	-0.0008	0.0000	0.0217	0.0184	0.0070	0.0202
	p	0.1307	0.2400	0.4188	0.9494	0.3306	0.3531	0.3574	0.5117	0.4886	0.5982	0.4042	0.0000	0.0006	0.0989	0.0082

Continue Table S8:

Loci		HebeiChengde-Manchu	Liaoning-Hui	Gansu-Hui	Yunnan-Bai	Yunnan-Vietnamese	Yunnan-Miao	Yunnan-Yi	Yunnan-Zhuang	Yunnan-Dai	Yunnan-Hani	Yili-Xibe	Liangshan-Tibetan	Liangshan-Yi
CSF1PO	Fst	-0.0007	-0.0011	-0.0011	-0.0002	0.0364	0.0452	0.0000	-0.0006	0.0050	-0.0045	0.0052	-0.0043	0.0087
	p	0.4759	0.5168	0.4911	0.4117	0.0000	0.0000	0.3878	0.4602	0.1586	0.9334	0.0968	0.9495	0.0478
D2S1338	Fst	-0.0002	0.0012	0.0012	0.0037	0.0089	0.0182	0.0039	0.0004	-0.0036	0.0046	0.0026	0.0049	0.0009
	p	0.4577	0.2766	0.2681	0.0309	0.0022	0.0000	0.0399	0.3576	0.8624	0.0788	0.1440	0.0622	0.3108
D3S1358	Fst	0.0064	0.0033	0.0001	0.0029	-0.0003	-0.0005	0.0086	-0.0035	-0.0052	-0.0035	0.0077	0.0112	0.0083
	p	0.0446	0.1570	0.3617	0.1164	0.4059	0.4474	0.0161	0.8653	0.8677	0.7849	0.0507	0.0273	0.0594
D5S818	Fst	-0.0003	-0.0034	0.0001	0.0018	0.0000	0.0026	-0.0030	-0.0017	-0.0004	-0.0038	-0.0008	-0.0025	-0.0048
	p	0.4315	0.8912	0.3852	0.1725	0.4087	0.1320	0.9734	0.6302	0.4286	0.8855	0.4980	0.7358	0.9873
D6S1043	Fst	0.0153	0.0113	0.0087	0.0128	0.0113	0.0142	0.0184	0.0147	0.0157	0.0118	0.0156	0.0190	0.0149
	p	0.0000	0.0004	0.0055	0.0000	0.0001	0.0000	0.0000	0.0001	0.0020	0.0025	0.0000	0.0000	0.0002
D7S820	Fst	-0.0012	0.0004	0.0016	0.0004	0.0140	0.0158	0.0029	-0.0023	0.0005	-0.0053	0.0078	0.0092	0.0050
	p	0.5943	0.3543	0.2373	0.3229	0.0014	0.0009	0.1140	0.7511	0.3520	0.9992	0.0371	0.0262	0.0895
D8S1179	Fst	0.0007	-0.0003	0.0113	-0.0006	0.0187	0.0359	0.0005	0.0013	0.0089	-0.0008	0.0052	0.0086	0.0004
	p	0.3167	0.4673	0.0034	0.5736	0.0000	0.0000	0.3369	0.2645	0.0301	0.5085	0.0565	0.0172	0.3719
D12S391	Fst	-0.0002	-0.0038	-0.0010	0.0017	0.0175	0.0325	0.0049	-0.0010	-0.0027	-0.0012	0.2085	0.0025	0.0380
	p	0.4556	0.9830	0.5731	0.1456	0.0000	0.0000	0.0261	0.5980	0.7275	0.5845	0.0000	0.1678	0.0000
D13S317	Fst	-0.0033	-0.0033	0.0020	-0.0019	0.0352	0.0691	-0.0008	-0.0003	-0.0024	-0.0035	-0.0032	0.0009	-0.0021
	p	0.9897	0.8814	0.2156	0.8439	0.0000	0.0000	0.5537	0.4352	0.6358	0.8568	0.8815	0.3057	0.6892
D16S539	Fst	-0.0015	-0.0036	-0.0043	-0.0020	0.0073	0.0529	-0.0017	0.0042	-0.0010	-0.0029	-0.0042	0.0147	-0.0001
	p	0.6495	0.9228	0.9889	0.8652	0.0233	0.0000	0.7369	0.1104	0.4764	0.7552	0.9793	0.0053	0.4133
D18S51	Fst	0.0002	-0.0027	-0.0034	-0.0010	0.0039	0.0081	-0.0012	-0.0016	-0.0049	0.0019	-0.0010	0.0094	0.0046
	p	0.3661	0.8985	0.9678	0.6770	0.0540	0.0041	0.6800	0.7010	0.9673	0.2073	0.5754	0.0116	0.0717
D19S433	Fst	-0.0011	-0.0030	0.0005	-0.0014	0.0131	0.0209	0.0047	-0.0020	-0.0059	-0.0013	-0.0024	-0.0020	-0.0037
	p	0.6193	0.9074	0.3408	0.7786	0.0010	0.0000	0.0374	0.7687	0.9950	0.5717	0.7905	0.7125	0.9518
D21S11	Fst	0.0000	-0.0029	-0.0023	-0.0015	0.0060	0.0117	-0.0010	-0.0018	-0.0038	-0.0022	-0.0009	-0.0021	-0.0031
	p	0.3888	0.8847	0.8080	0.8055	0.0167	0.0003	0.6247	0.7159	0.8596	0.7216	0.5283	0.7388	0.8852
FGA	Fst	0.0027	-0.0003	0.0022	0.0014	0.0218	0.0373	0.0021	0.0014	0.0001	-0.0036	0.0041	0.0052	0.0048
	p	0.1004	0.4535	0.1648	0.1626	0.0000	0.0000	0.1205	0.2362	0.4054	0.9492	0.0776	0.0429	0.0657

PentaD	Fst	0.0061	0.0072	0.0176	0.0013	0.0011	0.0013	0.0021	-0.0033	-0.0037	0.0015	0.0091	0.0186	0.0023
	p	0.0263	0.0280	0.0004	0.1958	0.2490	0.2129	0.1535	0.9406	0.8192	0.2564	0.0153	0.0006	0.1984
PentaE	Fst	-0.0021	-0.0030	0.0041	0.0003	0.0014	0.0799	0.0034	0.0109	0.0117	0.0041	-0.0013	-0.0015	0.0005
	p	0.9504	0.9812	0.0390	0.3475	0.1504	0.0000	0.0249	0.0009	0.0023	0.0558	0.7481	0.7676	0.3502
TH01	Fst	-0.0005	-0.0004	-0.0001	0.0012	0.0052	0.0107	0.0036	0.0082	-0.0008	-0.0019	0.0015	0.0003	-0.0005
	p	0.4449	0.4168	0.3916	0.2180	0.0580	0.0086	0.0978	0.0440	0.4406	0.5839	0.2514	0.3446	0.4078
TPOX	Fst	-0.0023	-0.0039	-0.0044	-0.0028	0.0561	0.0674	-0.0020	-0.0024	-0.0015	0.0021	-0.0035	0.0047	-0.0035
	p	0.7074	0.8899	0.9551	0.9639	0.0000	0.0000	0.6881	0.6391	0.4499	0.2417	0.8271	0.1356	0.7406
VWA	Fst	0.0032	0.0016	-0.0014	0.0019	0.0012	0.0068	-0.0019	-0.0025	-0.0035	0.0025	0.0039	0.0043	0.0113
	p	0.1129	0.2469	0.5819	0.1579	0.2467	0.0217	0.7850	0.7753	0.7515	0.2025	0.1106	0.1198	0.0141

Supplementary Table S9. The Fst and corresponding p value of locus-by-locus pairwise comparisons between the Sichuan Yi and other 58 relative reference populations.

Loci		Tibet-Tibetan	Sichuan-Han1	Sichuan-Han2	Sichuan-Han3	Chongqing-Han	GansuPingliang-Han	InnerMongolia-Han	Hunan-Han	Henan-Han1	Henan-Han2	Hubei-Han1	Hubei-Han2	Jiangxi-Han	Guangxi-Han	Guangdong-Han
CSF1PO	Fst	-0.0059	0.0028	0.0003	0.0006	0.0005	0.0016	-0.0011	0.0011	0.0026	0.0008	0.0003	0.0026	0.0025	-0.0013	0.0004
	p	0.8895	0.1342	0.3398	0.3207	0.3167	0.2547	0.5302	0.2570	0.1550	0.3143	0.3486	0.1198	0.1548	0.6006	0.3213
D2S1338	Fst	0.0001	-0.0023	-0.0019	-0.0023	-0.0027	-0.0028	-0.0018	-0.0018	-0.0024	-0.0030	-0.0021	-0.0018	-0.0021	0.0010	-0.0019
	p	0.4037	0.9267	0.9342	0.8663	0.9798	0.8757	0.8010	0.8549	0.9405	0.9387	0.8935	0.9028	0.8945	0.2315	0.9222
D3S1358	Fst	0.0105	0.0011	0.0032	-0.0009	0.0024	-0.0012	-0.0005	-0.0001	-0.0012	-0.0017	0.0005	0.0010	0.0015	0.0123	0.0022
	p	0.0691	0.2622	0.1000	0.4791	0.1683	0.5007	0.4360	0.3764	0.5443	0.5817	0.3183	0.2438	0.2261	0.0054	0.1620
D5S818	Fst	-0.0003	-0.0020	-0.0020	-0.0037	-0.0030	-0.0034	0.1208	-0.0028	-0.0021	-0.0014	-0.0018	-0.0016	-0.0018	-0.0021	-0.0026
	p	0.4158	0.7739	0.8759	0.9671	0.9796	0.8637	0.0000	0.9507	0.7994	0.5698	0.7287	0.7536	0.7169	0.8126	0.9682
D6S1043	Fst	-0.0025	-0.0013	-0.0020	-0.0023	-0.0008	0.0005	-0.0023	-0.0013	0.0188	-0.0012	-0.0019	-0.0013	-0.0016	-0.0022	-0.0022
	p	0.7118	0.7302	0.9457	0.8629	0.6014	0.3421	0.9053	0.7341	0.0000	0.6279	0.8702	0.7884	0.7748	0.9289	0.9573
D7S820	Fst	-0.0057	0.0032	0.0045	0.0002	0.0066	0.0005	0.0024	0.0042	0.0004	0.0000	0.0012	0.0041	0.0083	0.0159	0.0025
	p	0.9267	0.1013	0.0427	0.3747	0.0241	0.3425	0.1614	0.0650	0.3487	0.3933	0.2539	0.0448	0.0112	0.0005	0.1160
D8S1179	Fst	-0.0054	0.0010	0.0008	-0.0019	0.0014	-0.0027	-0.0010	0.0035	-0.0018	-0.0037	-0.0012	0.0000	0.0021	0.0116	0.0015
	p	0.9312	0.2560	0.2618	0.7546	0.2139	0.8080	0.6095	0.0622	0.7941	0.9725	0.6585	0.4116	0.1561	0.0005	0.1832
D12S391	Fst	0.0360	0.0417	0.0410	0.0432	0.0432	0.0421	0.0433	0.0394	0.0450	0.0407	0.0428	0.0405	0.0418	0.0385	0.0393
	p	0.0000	0.0000	0.0000	0.0000	0.0000	0.0000	0.0000	0.0000	0.0000	0.0000	0.0000	0.0000	0.0000	0.0000	0.0000
D13S317	Fst	-0.0045	0.0014	0.0003	-0.0011	0.0004	-0.0011	-0.0017	-0.0008	-0.0010	-0.0036	-0.0007	-0.0003	-0.0003	0.0023	-0.0017
	p	0.8282	0.2248	0.3481	0.5668	0.3421	0.5644	0.7032	0.5466	0.5884	0.9531	0.5262	0.4593	0.4495	0.1467	0.7918
D16S539	Fst	-0.0001	0.0007	0.0011	0.0037	0.0016	-0.0011	0.0805	-0.0001	0.0007	-0.0005	-0.0008	0.0012	0.0010	0.0001	-0.0004
	p	0.4093	0.2997	0.2388	0.1185	0.2116	0.5388	0.0000	0.4124	0.3072	0.4660	0.5360	0.2239	0.2648	0.3807	0.4778
D18S51	Fst	-0.0030	0.0075	0.0045	0.0032	0.0079	0.0042	0.0011	0.0079	0.0049	0.0009	0.0021	0.0043	0.0063	0.0181	0.0043
	p	0.7453	0.0056	0.0190	0.0966	0.0035	0.0849	0.2552	0.0025	0.0280	0.2878	0.1340	0.0240	0.0107	0.0000	0.0277
D19S433	Fst	-0.0037	-0.0028	-0.0020	-0.0025	-0.0017	-0.0019	-0.0017	-0.0020	-0.0018	-0.0016	-0.0024	-0.0018	-0.0022	-0.0020	-0.0013
	p	0.7982	0.9817	0.9348	0.8605	0.7827	0.6907	0.7275	0.8615	0.7974	0.6678	0.9185	0.8722	0.8700	0.8655	0.7291
D21S11	Fst	-0.0052	-0.0020	-0.0011	-0.0016	-0.0014	-0.0036	-0.0009	-0.0016	-0.0016	-0.0024	-0.0017	-0.0011	-0.0005	-0.0001	-0.0008
	p	0.9557	0.8746	0.6972	0.7022	0.7167	0.9603	0.5597	0.7611	0.7516	0.8371	0.7757	0.6789	0.4990	0.4225	0.5922
FGA	Fst	0.0026	0.0011	0.0013	0.0009	0.0021	0.0011	0.0088	0.0007	0.0003	-0.0017	0.0008	0.0004	0.0003	0.0054	0.0013
	p	0.2140	0.2271	0.1900	0.2694	0.1365	0.2786	0.0053	0.2852	0.3694	0.7097	0.2733	0.3158	0.3557	0.0122	0.1860

PentaD	Fst	0.0046	0.0011	-0.0003	-0.0029	-0.0002	-0.0010	0.0016	-0.0008	0.0003	0.0013	0.0009	0.0003	0.0014	0.0035	0.0014
	p	0.1499	0.2408	0.4555	0.8902	0.4294	0.5321	0.2035	0.5823	0.3624	0.2595	0.2802	0.3374	0.2219	0.0724	0.1962
PentaE	Fst	-0.0015	0.0007	0.0011	-0.0004	0.0012	0.0014	0.0181	0.0018	0.0002	0.0003	0.0009	0.0010	0.0008	0.0049	0.0004
	p	0.6429	0.2780	0.1673	0.5201	0.1848	0.2258	0.0000	0.1134	0.3902	0.3798	0.2389	0.1864	0.2542	0.0089	0.3207
TH01	Fst	-0.0032	-0.0015	-0.0018	-0.0038	-0.0026	-0.0041	-0.0025	-0.0024	-0.0022	-0.0032	-0.0014	-0.0014	-0.0021	-0.0001	-0.0022
	p	0.6184	0.6266	0.7580	0.9659	0.8739	0.9245	0.7725	0.8337	0.7703	0.8270	0.5780	0.6472	0.7347	0.3865	0.8548
TPOX	Fst	-0.0022	-0.0027	-0.0018	-0.0034	-0.0026	-0.0042	-0.0032	-0.0028	-0.0030	-0.0023	-0.0029	-0.0013	-0.0008	-0.0008	-0.0023
	p	0.4982	0.8297	0.6986	0.8508	0.7966	0.9052	0.8823	0.8710	0.8921	0.6132	0.8507	0.5857	0.4440	0.4752	0.8122
VWA	Fst	-0.0019	0.0065	0.0065	-0.0011	0.0058	0.0032	0.0026	0.0056	0.0024	-0.0001	0.0019	0.0045	0.0060	0.0136	0.0055
	p	0.4982	0.8297	0.6986	0.8508	0.7966	0.9052	0.8823	0.8710	0.8921	0.6132	0.8507	0.5857	0.4440	0.4752	0.8122

Continue Table S9:

Loci		Guangzhou-Han	Dongguan-Han	Foshan-Han	Jiangmen-Han-	Maoming-Han	Qingyuan-Han	Yangjiang-Han	Zhanjiang-Han	Zhaoqing-Han	Zhongshan-Han	Minnan-Han	Minxi-Han	Xiamen-Han	Beijing-Han	Tianjin-Han
CSF1PO	Fst	0.0009	0.0030	-0.0012	-0.0003	-0.0013	0.0004	0.0032	0.0048	-0.0005	-0.0005	0.0024	-0.0001	0.0032	0.0024	0.0018
	p	0.2579	0.1420	0.5981	0.4240	0.5583	0.3249	0.1048	0.0815	0.4587	0.4533	0.1837	0.3929	0.0908	0.2167	0.2565
D2S1338	Fst	-0.0006	-0.0012	-0.0012	0.0006	0.0015	0.0000	-0.0003	0.0004	0.0007	0.0000	-0.0020	-0.0006	-0.0019	-0.0031	-0.0033
	p	0.5769	0.6838	0.7285	0.3123	0.2060	0.4325	0.4859	0.3547	0.2793	0.4159	0.8091	0.5598	0.9427	0.8935	0.9143
D3S1358	Fst	0.0062	0.0054	0.0099	0.0102	0.0058	0.0077	0.0076	0.0055	0.0049	0.0048	0.0059	0.0016	0.0017	-0.0021	-0.0026
	p	0.0279	0.0734	0.0116	0.0084	0.0689	0.0191	0.0183	0.0699	0.0565	0.0745	0.0680	0.2188	0.1891	0.6049	0.6468
D5S818	Fst	-0.0017	-0.0020	-0.0020	-0.0019	-0.0004	-0.0015	-0.0019	-0.0017	-0.0010	-0.0017	-0.0037	-0.0024	-0.0016	-0.0050	-0.0038
	p	0.7947	0.7563	0.8102	0.8105	0.4459	0.7090	0.8317	0.6911	0.5964	0.7063	0.9871	0.8695	0.7677	0.9985	0.8839
D6S1043	Fst	-0.0019	-0.0028	-0.0020	-0.0018	-0.0028	0.0000	-0.0018	-0.0025	-0.0016	-0.0027	-0.0013	-0.0016	-0.0017	-0.0019	-0.0032
	p	0.9304	0.9786	0.9163	0.8870	0.9644	0.4183	0.9007	0.9342	0.8356	0.9844	0.6660	0.7994	0.8962	0.7268	0.9153
D7S820	Fst	0.0069	0.0083	0.0086	0.0092	0.0036	0.0025	0.0084	0.0072	0.0085	0.0056	0.0028	0.0049	0.0072	0.0034	0.0012
	p	0.0105	0.0151	0.0077	0.0067	0.1046	0.1316	0.0062	0.0231	0.0066	0.0352	0.1439	0.0517	0.0096	0.1524	0.2951
D8S1179	Fst	0.0065	0.0045	0.0080	0.0064	0.0089	0.0073	0.0072	0.0044	0.0080	0.0104	0.0028	0.0040	0.0013	-0.0023	-0.0042
	p	0.0037	0.0463	0.0025	0.0083	0.0043	0.0034	0.0031	0.0532	0.0013	0.0012	0.1215	0.0492	0.1974	0.7487	0.9760
D12S391	Fst	0.0368	0.0363	0.0380	0.0393	0.0355	0.0407	0.0369	0.0388	0.0356	0.0375	0.0418	0.0388	0.0408	0.0398	0.0439
	p	0.0000	0.0000	0.0000	0.0000	0.0000	0.0000	0.0000	0.0000	0.0000	0.0000	0.0000	0.0000	0.0000	0.0000	0.0000
D13S317	Fst	0.0013	-0.0001	0.0015	0.0037	0.0002	0.0028	0.0010	0.0008	0.0013	0.0009	-0.0015	-0.0006	-0.0005	-0.0035	-0.0041
	p	0.2002	0.4238	0.2065	0.0709	0.3650	0.1064	0.2551	0.2962	0.2217	0.2732	0.6221	0.4991	0.5041	0.8841	0.9496

D16S539	Fst	0.0004	0.0010	0.0013	0.0000	-0.0003	-0.0008	-0.0004	0.0023	0.0005	0.0014	0.0038	0.0007	0.0015	0.0008	0.0049
	p	0.3302	0.2708	0.2287	0.3965	0.4468	0.5429	0.4646	0.1683	0.3086	0.2206	0.1083	0.2979	0.2001	0.3181	0.0995
D18S51	Fst	0.0087	0.0081	0.0101	0.0105	0.0107	0.0078	0.0094	0.0115	0.0121	0.0124	0.0043	0.0050	0.0040	-0.0013	-0.0004
	p	0.0005	0.0050	0.0003	0.0007	0.0004	0.0015	0.0004	0.0008	0.0000	0.0001	0.0532	0.0221	0.0195	0.6117	0.4716
D19S433	Fst	-0.0019	-0.0024	-0.0021	-0.0023	-0.0025	-0.0007	-0.0014	-0.0025	-0.0016	-0.0024	-0.0009	-0.0017	-0.0016	-0.0021	-0.0036
	p	0.9076	0.9000	0.8961	0.9586	0.9124	0.5589	0.7681	0.9003	0.7967	0.9289	0.5565	0.7818	0.8471	0.7058	0.9285
D21S11	Fst	-0.0002	0.0002	0.0010	-0.0001	-0.0003	-0.0009	0.0002	-0.0005	0.0008	0.0000	0.0001	-0.0019	-0.0012	-0.0007	-0.0043
	p	0.4505	0.3703	0.2470	0.4223	0.4486	0.6175	0.3584	0.4865	0.2624	0.4017	0.3972	0.8473	0.7549	0.4979	0.9935
FGA	Fst	0.0042	0.0034	0.0059	0.0025	0.0048	0.0037	0.0062	0.0042	0.0060	0.0020	0.0029	0.0008	0.0010	-0.0015	-0.0014
	p	0.0198	0.0691	0.0104	0.0819	0.0375	0.0380	0.0063	0.0451	0.0052	0.1410	0.1033	0.2873	0.2210	0.6369	0.6279
PentaD	Fst	0.0025	0.0014	0.0038	0.0026	0.0042	0.0033	0.0022	0.0046	0.0029	0.0024	0.0005	0.0040	0.0015	0.0007	0.0004
	p	0.0897	0.2233	0.0588	0.0979	0.0665	0.0726	0.1223	0.0560	0.0865	0.1313	0.3402	0.0641	0.1683	0.3261	0.3612
PentaE	Fst	0.0026	0.0020	0.0028	0.0024	0.0026	0.0029	0.0025	0.0043	0.0035	0.0015	0.0016	0.0012	0.0013	0.0011	-0.0017
	p	0.0384	0.1156	0.0415	0.0579	0.0840	0.0392	0.0480	0.0220	0.0205	0.1567	0.1735	0.1867	0.1383	0.2768	0.7595
TH01	Fst	-0.0012	-0.0024	0.0002	-0.0005	-0.0025	0.0001	-0.0009	-0.0016	0.0019	-0.0016	0.0002	-0.0012	-0.0011	-0.0015	-0.0011
	p	0.6259	0.7916	0.3422	0.4541	0.7812	0.3627	0.5274	0.6157	0.1749	0.6585	0.3483	0.5778	0.5980	0.5149	0.4580
TPOX	Fst	0.0004	0.0015	0.0012	0.0013	0.0001	0.0010	0.0000	-0.0007	0.0010	0.0016	-0.0031	-0.0025	-0.0004	-0.0040	-0.0044
	p	0.3013	0.2320	0.2474	0.2321	0.3381	0.2590	0.3542	0.4279	0.2506	0.2198	0.8171	0.7762	0.4094	0.8519	0.8935
VWA	Fst	0.0093	0.0148	0.0080	0.0088	0.0114	0.0128	0.0110	0.0151	0.0137	0.0113	0.0009	0.0037	0.0051	0.0014	0.0031
	p	0.3013	0.2320	0.2474	0.2321	0.3381	0.2590	0.3542	0.4279	0.2506	0.2198	0.8171	0.7762	0.4094	0.8519	0.8935

Continue Table S9:

Loci		Shanghai-Han	Jiangsu-Huai'an-Han	Jiangsu-Yangzhou-Han	Jiangsu-Taizhou-Han	Anhui-Suzhou-Han	Shangdong-Han	Shanxi-Han	Jiangxi-Jiujiang-Han	Yunnan-Han	Yungui-Han	Taiwan-Han	Xinjiang-Uyghur1	Xinjiang-Uyghur2	Xinjiang-Uyghur3	Xinjiang-Kazakh
CSF1PO	Fst	0.0006	-0.0023	0.0017	-0.0012	-0.0018	0.0010	0.0030	0.0009	0.0024	0.0021	0.0039	0.0147	0.0046	0.0072	0.0025
	p	0.3011	0.5904	0.2699	0.4596	0.5786	0.3025	0.1337	0.2551	0.1444	0.1484	0.0764	0.0009	0.1265	0.1226	0.2647
D2S1338	Fst	-0.0028	-0.0030	-0.0039	-0.0041	-0.0028	-0.0046	-0.0024	-0.0017	-0.0020	-0.0021	-0.0015	-0.0003	0.0010	-0.0019	-0.0005
	p	0.9875	0.7952	0.9594	0.8885	0.8594	0.9961	0.9311	0.9009	0.9316	0.9513	0.8389	0.4873	0.3032	0.6270	0.4610
D3S1358	Fst	0.0011	-0.0051	-0.0026	0.0011	-0.0033	-0.0035	0.0007	0.0015	0.0012	0.0041	0.0024	0.0071	0.0001	0.0109	0.0021
	p	0.2565	0.8926	0.6477	0.3025	0.7699	0.7970	0.3021	0.1995	0.2294	0.0722	0.1467	0.0214	0.3627	0.0625	0.2802
D5S818	Fst	-0.0025	-0.0061	-0.0035	-0.0042	-0.0043	-0.0051	-0.0023	-0.0022	-0.0022	-0.0023	-0.0023	0.0051	0.0068	0.0015	-0.0050
	p	0.9003	0.9939	0.8272	0.7838	0.9526	0.9988	0.8249	0.9320	0.8971	0.9343	0.9219	0.0456	0.0616	0.3088	0.7827

D6S1043	Fst	-0.0009	-0.0042	-0.0034	0.0007	-0.0032	-0.0018	-0.0009	-0.0012	-0.0012	-0.0016	-0.0022	0.0072	0.0038	0.0001	0.0103
	p	0.6178	0.9449	0.9234	0.3640	0.9265	0.7182	0.6271	0.7574	0.7480	0.8669	0.9727	0.0034	0.0946	0.4119	0.0355
D7S820	Fst	0.0003	0.0039	-0.0028	-0.0016	0.0069	0.0026	0.0015	0.0057	0.0039	0.0034	0.0081	0.0043	0.0007	0.0609	0.0023
	p	0.3532	0.1650	0.7557	0.5338	0.0532	0.1899	0.2253	0.0179	0.0577	0.0728	0.0072	0.0501	0.3322	0.0000	0.2676
D8S1179	Fst	-0.0011	-0.0033	-0.0021	-0.0010	-0.0033	-0.0007	-0.0025	0.0011	-0.0002	0.0013	0.0030	0.0008	-0.0024	-0.0050	-0.0047
	p	0.6382	0.7959	0.6929	0.5131	0.8947	0.4994	0.9217	0.2168	0.4611	0.2013	0.0649	0.2763	0.7626	0.9082	0.8195
D12S391	Fst	0.0408	0.0419	0.0359	0.0328	0.0359	0.0453	0.0325	0.0396	0.0428	0.0432	0.0392	0.0423	0.0359	0.0233	0.0449
	p	0.0000	0.0000	0.0000	0.0000	0.0000	0.0000	0.0000	0.0000	0.0000	0.0000	0.0000	0.0000	0.0000	0.0003	0.0000
D13S317	Fst	-0.0006	-0.0008	-0.0005	-0.0056	-0.0026	-0.0009	-0.0024	-0.0003	-0.0012	-0.0006	-0.0007	0.0114	0.0217	0.0009	-0.0021
	p	0.5031	0.4649	0.4688	0.9535	0.7406	0.5058	0.8709	0.4663	0.6742	0.5275	0.5594	0.0008	0.0001	0.3434	0.5656
D16S539	Fst	-0.0011	-0.0011	0.0035	-0.0046	0.0044	0.0052	0.0017	0.0009	-0.0001	0.0021	0.0020	-0.0018	-0.0010	-0.0039	-0.0063
	p	0.5767	0.4920	0.1575	0.8479	0.1109	0.0890	0.2056	0.2532	0.4175	0.1399	0.1609	0.7766	0.5271	0.7637	0.9008
D18S51	Fst	0.0036	-0.0005	-0.0008	0.0013	-0.0010	-0.0021	0.0038	0.0037	0.0044	0.0035	0.0119	0.0045	0.0043	0.0061	0.0017
	p	0.0545	0.4575	0.5174	0.2922	0.5700	0.7329	0.0570	0.0316	0.0193	0.0417	0.0001	0.0239	0.0757	0.0868	0.2888
D19S433	Fst	-0.0016	-0.0025	-0.0023	-0.0050	-0.0025	-0.0029	-0.0022	-0.0016	-0.0018	-0.0018	-0.0013	0.0050	0.0065	0.0009	0.0158
	p	0.7494	0.6965	0.7254	0.9367	0.7704	0.8465	0.8703	0.8476	0.8896	0.8934	0.7289	0.0268	0.0430	0.3350	0.0173
D21S11	Fst	-0.0016	-0.0008	-0.0009	-0.0040	-0.0005	-0.0030	-0.0017	-0.0012	-0.0010	-0.0008	-0.0002	0.0002	0.0004	-0.0038	-0.0025
	p	0.7658	0.4947	0.5091	0.8593	0.4693	0.8705	0.7710	0.7396	0.6438	0.5856	0.4490	0.3703	0.3576	0.8368	0.6377
FGA	Fst	0.0000	-0.0034	-0.0024	-0.0039	0.0018	-0.0022	-0.0005	0.0010	0.0007	0.0006	0.0029	0.0085	0.0029	0.0012	-0.0046
	p	0.4126	0.8447	0.7800	0.8553	0.2118	0.7564	0.5038	0.2100	0.2780	0.2790	0.0631	0.0014	0.1400	0.3066	0.8378
PentaD	Fst	0.0009	0.0025	0.0096	0.0011	0.0002	-0.0013	-0.0011	0.0011	0.0001	0.0001	0.0033	0.0034	0.0090	-0.0033	0.0074
	p	0.2688	0.2217	0.0188	0.3229	0.3750	0.5646	0.6044	0.2168	0.3940	0.3786	0.0635	0.0657	0.0184	0.7269	0.0976
PentaE	Fst	0.0011	0.0006	-0.0027	0.0013	-0.0003	-0.0011	0.0000	0.0010	0.0009	0.0005	0.0018	0.0049	0.0078	0.0068	0.0039
	p	0.1975	0.3465	0.9009	0.2921	0.5022	0.6549	0.4377	0.1686	0.2052	0.2896	0.0970	0.0030	0.0030	0.0265	0.1373
TH01	Fst	-0.0012	-0.0019	-0.0012	0.0058	-0.0040	-0.0016	-0.0017	-0.0016	-0.0014	-0.0022	0.0001	0.0325	0.0459	0.0325	0.0441
	p	0.5464	0.5231	0.4609	0.1342	0.8926	0.5151	0.6343	0.7213	0.6364	0.8645	0.3600	0.0000	0.0000	0.0021	0.0006
TPOX	Fst	-0.0019	-0.0015	-0.0037	-0.0046	-0.0040	-0.0043	-0.0029	-0.0017	-0.0010	-0.0020	0.0028	0.0007	-0.0011	-0.0029	0.0094
	p	0.6512	0.4662	0.7662	0.7540	0.8388	0.8847	0.8765	0.7064	0.5255	0.7386	0.1292	0.2829	0.4562	0.5653	0.1124
VWA	Fst	-0.0002	-0.0019	-0.0003	0.0068	0.0007	-0.0010	0.0013	0.0045	0.0041	0.0056	0.0034	0.0010	0.0017	0.0015	0.0003
	p	0.6512	0.4662	0.7662	0.7540	0.8388	0.8847	0.8765	0.7064	0.5255	0.7386	0.1292	0.2829	0.4562	0.5653	0.1124

Continue Table S9:

Loci		HebeiChengde-Manchu	Liaoning-Hui	Gansu-Hui	Yunnan-Bai	Yunnan-Vietnamese	Yunnan-Miao	Yunnan-Yi	Yunnan-Zhuang	Yunnan-Dai	Yunnan-Hani	Yili-Xibe	Liangshan-Tibatan	Hainan-Han
CSF1PO	Fst	0.0014	0.0075	0.0039	0.0021	0.0062	0.0096	0.0013	-0.0001	-0.0053	0.0076	-0.0012	0.0079	0.0087
	p	0.2398	0.0619	0.1449	0.1636	0.0534	0.0166	0.2446	0.3896	0.8892	0.0728	0.5069	0.0597	0.0478
D2S1338	Fst	-0.0031	-0.0019	-0.0033	-0.0020	0.0065	0.0155	-0.0011	0.0037	0.0004	-0.0027	-0.0039	-0.0022	0.0009
	p	0.9844	0.7110	0.9427	0.9143	0.0125	0.0002	0.6777	0.0976	0.3851	0.8090	0.9858	0.7550	0.3108
D3S1358	Fst	-0.0017	-0.0008	0.0072	-0.0002	0.0254	0.0236	0.0007	0.0055	0.0083	0.0053	-0.0044	-0.0042	0.0083
	p	0.6154	0.4632	0.0628	0.4015	0.0004	0.0001	0.2922	0.1007	0.0986	0.1186	0.9455	0.8823	0.0594
D5S818	Fst	-0.0022	-0.0044	-0.0024	-0.0003	-0.0018	0.0008	-0.0026	-0.0019	0.0009	-0.0037	-0.0012	-0.0038	-0.0048
	p	0.7647	0.9777	0.7122	0.4323	0.7007	0.2782	0.8866	0.6518	0.3370	0.8540	0.5324	0.8928	0.9873
D6S1043	Fst	-0.0022	-0.0029	-0.0010	-0.0016	-0.0001	0.0022	-0.0008	-0.0012	-0.0001	0.0009	-0.0027	-0.0040	0.0149
	p	0.8889	0.9056	0.5891	0.8502	0.4417	0.1099	0.5857	0.6268	0.4304	0.3233	0.8817	0.9861	0.0002
D7S820	Fst	0.0038	-0.0018	-0.0006	0.0006	0.0400	0.0419	0.0033	0.0143	0.0213	0.0030	-0.0009	-0.0030	0.0050
	p	0.1021	0.6449	0.4722	0.3096	0.0000	0.0000	0.1059	0.0041	0.0049	0.1763	0.4926	0.7968	0.0895
D8S1179	Fst	-0.0004	-0.0036	0.0009	-0.0016	0.0328	0.0498	0.0066	0.0119	0.0130	0.0021	0.0007	-0.0001	0.0004
	p	0.4766	0.9378	0.3058	0.7912	0.0000	0.0000	0.0122	0.0017	0.0113	0.2255	0.3240	0.4297	0.3719
D12S391	Fst	0.0477	0.0376	0.0431	0.0463	0.0537	0.0695	0.0417	0.0305	0.0489	0.0468	0.2076	0.0391	0.0380
	p	0.0000	0.0000	0.0000	0.0000	0.0000	0.0000	0.0000	0.0000	0.0000	0.0000	0.0000	0.0000	0.0000
D13S317	Fst	-0.0024	-0.0039	-0.0028	-0.0007	0.0362	0.0702	-0.0010	0.0013	0.0034	0.0002	-0.0040	-0.0025	-0.0021
	p	0.8461	0.9511	0.8106	0.5395	0.0000	0.0000	0.5801	0.2779	0.1928	0.3909	0.9555	0.7416	0.6892
D16S539	Fst	0.0038	-0.0003	0.0004	0.0003	0.0134	0.0779	-0.0003	-0.0003	-0.0046	-0.0006	-0.0011	0.0188	-0.0001
	p	0.0992	0.4213	0.3567	0.3513	0.0031	0.0000	0.4446	0.4269	0.8506	0.4570	0.5353	0.0024	0.4133
D18S51	Fst	-0.0008	-0.0004	0.0074	0.0062	0.0077	0.0123	0.0093	0.0129	0.0088	-0.0011	-0.0015	0.0017	0.0046
	p	0.5498	0.4628	0.0186	0.0097	0.0069	0.0007	0.0040	0.0012	0.0353	0.5770	0.6537	0.2266	0.0717
D19S433	Fst	-0.0014	-0.0046	-0.0010	-0.0011	0.0149	0.0229	0.0037	-0.0020	-0.0045	-0.0015	-0.0026	-0.0016	-0.0037
	p	0.6578	0.9996	0.5413	0.6765	0.0003	0.0000	0.0681	0.7387	0.9006	0.5916	0.8210	0.6414	0.9518
D21S11	Fst	-0.0002	-0.0010	-0.0029	-0.0016	0.0035	0.0083	-0.0012	-0.0017	-0.0045	-0.0029	-0.0021	-0.0030	-0.0031
	p	0.4284	0.5489	0.8880	0.8111	0.0797	0.0040	0.6637	0.7100	0.9311	0.8295	0.7280	0.8842	0.8852
FGA	Fst	-0.0004	-0.0019	0.0005	-0.0001	0.0233	0.0369	0.0110	0.0132	0.0142	0.0066	-0.0020	0.0013	0.0048
	p	0.4872	0.7343	0.3421	0.4468	0.0000	0.0000	0.0001	0.0009	0.0065	0.0408	0.7444	0.2678	0.0657

PentaD	Fst	0.0008	0.0012	0.0040	-0.0002	0.0060	0.0057	-0.0008	0.0063	0.0107	0.0002	0.0008	0.0069	0.0023
	p	0.2850	0.2703	0.1107	0.4533	0.0261	0.0277	0.5534	0.0457	0.0325	0.3758	0.3108	0.0480	0.1984
PentaE	Fst	-0.0014	-0.0019	0.0010	0.0009	0.0043	0.0841	0.0062	0.0109	0.0128	0.0074	0.0052	-0.0017	0.0005
	p	0.7970	0.8219	0.2740	0.2355	0.0178	0.0000	0.0013	0.0019	0.0038	0.0146	0.0187	0.7645	0.3502
TH01	Fst	-0.0009	-0.0037	0.0031	0.0000	0.0051	0.0128	-0.0013	0.0017	-0.0068	-0.0040	-0.0001	-0.0022	-0.0005
	p	0.4863	0.8712	0.1680	0.3586	0.0716	0.0055	0.5629	0.2313	0.9957	0.8544	0.3855	0.6262	0.4078
TPOX	Fst	-0.0037	-0.0046	-0.0025	-0.0021	0.0453	0.0544	0.0006	-0.0002	0.0019	0.0081	-0.0043	0.0109	-0.0035
	p	0.9686	0.9659	0.6226	0.7307	0.0000	0.0000	0.2952	0.3743	0.2624	0.0768	0.9275	0.0405	0.7406
VWA	Fst	-0.0011	0.0038	0.0014	0.0031	0.0269	0.0392	0.0091	0.0164	0.0060	-0.0020	0.0014	-0.0007	0.0113
	p	0.9686	0.9659	0.6226	0.7307	0.0000	0.0000	0.2952	0.3743	0.2624	0.0768	0.9275	0.0405	0.7406

Supplementary Table S10. The Fst and corresponding p value of locus-by-locus pairwise comparisons between the Sichuan Tibetan and other 58 relative reference populations.

Loci		Tibet-Tibetan	Sichuan - Han1	Sichuan-Han2	Sichuan - Han3	Chongqing -Han	GansuPingliang -Han	InnerMongolia -Han	Hunan-Han	Henan-Han1	Henan-Han2	Hubei-Han1	Hubei-Han2	Jiangxi-Han	Guangxi-Han	Guangdong-Han
CSF1PO	Fst	0.0025	0.0011	0.0028	-0.0002	0.0012	-0.0024	0.0007	0.0052	0.0005	-0.0013	-0.0003	0.0003	0.0001	0.0019	0.0021
	p	0.2507	0.2501	0.0993	0.4030	0.2291	0.6861	0.3071	0.0466	0.3151	0.5480	0.4244	0.3398	0.3633	0.1817	0.1549
D2S1338	Fst	-0.0012	0.0002	-0.0001	0.0004	-0.0003	0.0006	0.0025	-0.0004	0.0017	-0.0016	0.0002	0.0011	0.0006	0.0033	0.0007
	p	0.5341	0.3689	0.4335	0.3428	0.4889	0.3327	0.1098	0.5140	0.1555	0.7377	0.3820	0.1977	0.2986	0.0456	0.2649
D3S1358	Fst	0.0142	0.0025	0.0050	-0.0002	0.0030	0.0011	0.0024	0.0015	0.0022	0.0008	0.0027	0.0032	0.0051	0.0129	0.0026
	p	0.0366	0.1517	0.0409	0.3938	0.1310	0.2845	0.1617	0.2162	0.1756	0.2980	0.1486	0.0833	0.0629	0.0028	0.1317
D5S818	Fst	-0.0017	-0.0023	-0.0006	-0.0033	-0.0012	-0.0019	0.1241	-0.0018	-0.0020	-0.0012	-0.0006	-0.0018	-0.0023	0.0005	-0.0011
	p	0.5336	0.8860	0.5417	0.9494	0.6521	0.6625	0.0000	0.7814	0.8037	0.5695	0.5008	0.8610	0.8808	0.3248	0.6447
D6S1043	Fst	0.0012	0.0013	-0.0005	-0.0006	0.0009	0.0008	-0.0008	-0.0004	0.0215	-0.0003	-0.0005	-0.0001	0.0001	-0.0004	-0.0006
	p	0.3128	0.1913	0.5743	0.5315	0.2553	0.3093	0.5962	0.5170	0.0000	0.4707	0.5463	0.4592	0.4022	0.5214	0.6080
D7S820	Fst	-0.0020	0.0072	0.0090	0.0037	0.0123	0.0065	0.0064	0.0088	0.0030	0.0022	0.0045	0.0085	0.0125	0.0209	0.0051
	p	0.5733	0.0144	0.0022	0.0991	0.0011	0.0514	0.0254	0.0059	0.1059	0.1886	0.0550	0.0032	0.0016	0.0001	0.0277
D8S1179	Fst	0.0039	0.0109	0.0088	0.0050	0.0092	-0.0004	0.0060	0.0107	0.0026	0.0011	0.0058	0.0072	0.0124	0.0222	0.0087
	p	0.1740	0.0003	0.0002	0.0479	0.0017	0.4528	0.0188	0.0003	0.1092	0.2754	0.0173	0.0019	0.0002	0.0000	0.0004
D12S391	Fst	-0.0060	0.0010	0.0009	-0.0018	0.0002	-0.0017	-0.0010	0.0008	-0.0018	-0.0019	-0.0015	0.0018	0.0019	0.0068	0.0009
	p	0.9875	0.2435	0.2367	0.7703	0.3592	0.6912	0.6244	0.2650	0.8172	0.7456	0.7419	0.1311	0.1506	0.0052	0.2433
D13S317	Fst	-0.0036	0.0040	0.0035	-0.0001	0.0026	0.0015	-0.0011	0.0025	0.0001	-0.0030	0.0005	0.0018	0.0021	0.0050	0.0008
	p	0.7553	0.0592	0.0534	0.4159	0.1171	0.2461	0.6050	0.1206	0.3782	0.9057	0.3183	0.1377	0.1567	0.0349	0.2592
D16S539	Fst	-0.0012	0.0176	0.0165	0.0327	0.0203	0.0149	0.1350	0.0174	0.0218	0.0150	0.0211	0.0192	0.0197	0.0089	0.0173
	p	0.4999	0.0001	0.0000	0.0000	0.0000	0.0046	0.0000	0.0001	0.0000	0.0022	0.0000	0.0000	0.0000	0.0067	0.0000
D18S51	Fst	0.0015	0.0139	0.0101	0.0100	0.0115	0.0105	0.0063	0.0132	0.0072	0.0055	0.0064	0.0100	0.0085	0.0241	0.0082
	p	0.2713	0.0001	0.0001	0.0045	0.0006	0.0058	0.0142	0.0000	0.0061	0.0381	0.0097	0.0001	0.0021	0.0000	0.0006
D19S433	Fst	-0.0054	0.0013	0.0017	-0.0004	-0.0016	-0.0017	-0.0007	0.0009	0.0005	0.0022	0.0010	0.0020	0.0030	0.0012	0.0019
	p	0.9672	0.2055	0.1353	0.4816	0.8073	0.7028	0.5181	0.2581	0.3283	0.1645	0.2512	0.1134	0.0907	0.2180	0.1275
D21S11	Fst	-0.0031	0.0003	0.0005	-0.0011	0.0006	-0.0007	0.0014	0.0003	0.0000	-0.0010	0.0006	0.0004	0.0002	0.0012	0.0002
	p	0.7551	0.3319	0.2997	0.6189	0.3038	0.5100	0.2056	0.3534	0.3937	0.5706	0.2939	0.3158	0.3673	0.2182	0.3432
FGA	Fst	-0.0018	0.0017	0.0008	0.0003	-0.0007	-0.0001	0.0075	0.0004	-0.0014	0.0002	-0.0005	0.0002	-0.0006	0.0013	0.0002
	p	0.6331	0.1484	0.2342	0.3659	0.5984	0.4228	0.0049	0.3269	0.7593	0.3747	0.5250	0.3714	0.5569	0.1824	0.3693

PentaD	Fst	-0.0042	0.0087	0.0087	0.0082	0.0067	0.0014	0.0092	0.0073	0.0077	0.0081	0.0085	0.0100	0.0093	0.0167	0.0081
	p	0.8317	0.0046	0.0015	0.0140	0.0110	0.2510	0.0045	0.0071	0.0074	0.0177	0.0054	0.0008	0.0046	0.0001	0.0049
PentaE	Fst	-0.0057	-0.0002	0.0007	-0.0024	-0.0001	0.0022	0.0151	0.0006	-0.0014	-0.0016	-0.0004	-0.0003	-0.0001	0.0062	-0.0003
	p	0.9992	0.4838	0.2301	0.9639	0.4579	0.1354	0.0000	0.2622	0.8742	0.8305	0.5566	0.5273	0.4514	0.0013	0.5308
TH01	Fst	-0.0029	-0.0019	-0.0011	-0.0014	-0.0013	-0.0013	-0.0013	0.0015	-0.0010	-0.0027	-0.0014	-0.0005	-0.0004	0.0061	0.0004
	p	0.6037	0.7450	0.6072	0.5629	0.5915	0.5045	0.5661	0.1959	0.5429	0.7720	0.6031	0.4626	0.4204	0.0358	0.3132
TPOX	Fst	0.0207	0.0117	0.0100	0.0061	0.0113	0.0159	0.0118	0.0105	0.0146	0.0200	0.0117	0.0077	0.0064	0.0131	0.0087
	p	0.0203	0.0101	0.0099	0.0748	0.0118	0.0121	0.0148	0.0114	0.0050	0.0032	0.0119	0.0202	0.0485	0.0077	0.0181
VWA	Fst	-0.0042	0.0029	0.0021	-0.0021	0.0014	-0.0035	0.0009	0.0027	0.0015	-0.0007	0.0004	0.0015	0.0005	0.0067	0.0011
	p	0.8029	0.0983	0.1278	0.7588	0.2125	0.9190	0.2796	0.1199	0.2068	0.5117	0.3366	0.1747	0.3213	0.0147	0.2268

Continue Table S10:

Loci		Guangzhou-Han	Dongguan-Han	Foshan-Han	Jiangmen-Han	Maoming-Han	Qingyuan-Han	Yangjiang-Han	Zhanjiang-Han	Zhaoqing-Han	Zhongshan-Han	Minnan-Han	Minxi-Han	Xiamen-Han	Beijing-Han	Tianjin-Han
CSF1PO	Fst	0.0005	-0.0015	0.0021	0.0012	0.0004	0.0009	-0.0012	-0.0007	0.0020	0.0013	-0.0015	0.0008	-0.0001	0.0036	-0.0029
	p	0.2953	0.6281	0.1593	0.2265	0.3408	0.2571	0.6389	0.4816	0.1485	0.2299	0.6057	0.2797	0.3998	0.1577	0.7615
D2S1338	Fst	0.0026	-0.0003	0.0030	0.0034	0.0042	0.0033	0.0032	0.0027	0.0023	0.0045	-0.0025	0.0022	0.0003	-0.0020	-0.0012
	p	0.0537	0.4727	0.0534	0.0367	0.0404	0.0393	0.0412	0.0984	0.0839	0.0242	0.9228	0.1077	0.3408	0.7484	0.6097
D3S1358	Fst	0.0073	0.0080	0.0091	0.0119	0.0075	0.0095	0.0086	0.0087	0.0060	0.0070	0.0107	0.0039	0.0042	-0.0006	0.0007
	p	0.0107	0.0275	0.0089	0.0025	0.0318	0.0061	0.0090	0.0206	0.0321	0.0283	0.0140	0.0885	0.0547	0.4306	0.3169
D5S818	Fst	0.0003	0.0003	-0.0003	0.0007	0.0012	0.0006	-0.0004	0.0011	0.0014	-0.0004	-0.0014	-0.0005	-0.0006	-0.0040	-0.0030
	p	0.3354	0.3464	0.4431	0.2713	0.2573	0.2934	0.4804	0.2607	0.1979	0.4624	0.6407	0.4812	0.5519	0.9444	0.8098
D6S1043	Fst	-0.0003	-0.0016	-0.0005	0.0001	-0.0014	0.0019	0.0003	-0.0001	0.0002	-0.0013	0.0012	-0.0001	0.0000	-0.0007	-0.0014
	p	0.5094	0.8069	0.5526	0.4149	0.7557	0.1088	0.3633	0.4462	0.3762	0.7676	0.2425	0.4379	0.4282	0.5353	0.6602
D7S820	Fst	0.0115	0.0128	0.0127	0.0139	0.0078	0.0060	0.0139	0.0118	0.0132	0.0092	0.0066	0.0099	0.0118	0.0066	0.0052
	p	0.0006	0.0016	0.0011	0.0001	0.0159	0.0155	0.0001	0.0031	0.0005	0.0054	0.0323	0.0040	0.0004	0.0560	0.0848
D8S1179	Fst	0.0165	0.0154	0.0182	0.0156	0.0157	0.0165	0.0173	0.0102	0.0187	0.0230	0.0087	0.0146	0.0092	-0.0012	0.0039
	p	0.0000	0.0001	0.0000	0.0000	0.0000	0.0000	0.0000	0.0007	0.0000	0.0000	0.0056	0.0000	0.0002	0.5771	0.1099
D12S391	Fst	0.0021	0.0004	0.0029	0.0030	0.0007	0.0038	0.0030	0.0027	0.0038	0.0021	0.0040	0.0008	0.0008	-0.0020	-0.0037
	p	0.0911	0.3396	0.0720	0.0611	0.2964	0.0337	0.0539	0.1031	0.0333	0.1285	0.0608	0.2764	0.2511	0.7230	0.9569
D13S317	Fst	0.0058	0.0030	0.0060	0.0078	0.0066	0.0081	0.0066	0.0066	0.0058	0.0060	0.0006	0.0019	0.0026	-0.0025	-0.0018
	p	0.0129	0.1021	0.0176	0.0051	0.0209	0.0040	0.0094	0.0203	0.0176	0.0225	0.3308	0.1684	0.0857	0.7548	0.6509

D16S539	Fst	0.0103	0.0090	0.0084	0.0094	0.0183	0.0141	0.0132	0.0054	0.0105	0.0087	0.0169	0.0158	0.0176	0.0146	0.0324
	p	0.0010	0.0094	0.0056	0.0029	0.0002	0.0003	0.0003	0.0459	0.0018	0.0071	0.0010	0.0005	0.0000	0.0057	0.0000
D18S51	Fst	0.0139	0.0131	0.0180	0.0152	0.0188	0.0129	0.0163	0.0185	0.0186	0.0166	0.0114	0.0105	0.0088	-0.0004	0.0056
	p	0.0000	0.0005	0.0000	0.0000	0.0000	0.0001	0.0000	0.0000	0.0000	0.0000	0.0018	0.0003	0.0001	0.4597	0.0510
D19S433	Fst	0.0023	0.0020	0.0032	0.0015	0.0002	0.0028	0.0029	0.0004	0.0005	0.0028	0.0068	0.0010	0.0021	0.0028	-0.0014
	p	0.0928	0.1576	0.0622	0.1738	0.3739	0.0727	0.0723	0.3401	0.2923	0.0926	0.0194	0.2436	0.0969	0.1549	0.6341
D21S11	Fst	0.0010	0.0011	0.0035	0.0013	-0.0004	-0.0008	0.0022	0.0006	0.0018	0.0017	0.0003	-0.0011	0.0002	-0.0008	-0.0032
	p	0.2080	0.2323	0.0512	0.1892	0.4650	0.5982	0.1102	0.3025	0.1427	0.1563	0.3555	0.6538	0.3570	0.5125	0.9179
FGA	Fst	0.0036	-0.0001	0.0038	0.0023	0.0033	0.0027	0.0040	0.0022	0.0028	0.0025	-0.0008	0.0001	0.0007	-0.0026	-0.0011
	p	0.0211	0.4297	0.0315	0.0728	0.0648	0.0657	0.0206	0.1286	0.0571	0.0902	0.5948	0.3998	0.2375	0.8688	0.5963
PentaD	Fst	0.0158	0.0133	0.0150	0.0131	0.0165	0.0178	0.0133	0.0217	0.0146	0.0160	0.0087	0.0142	0.0117	0.0048	0.0028
	p	0.0001	0.0007	0.0001	0.0003	0.0003	0.0000	0.0003	0.0000	0.0003	0.0001	0.0092	0.0003	0.0006	0.0780	0.1706
PentaE	Fst	0.0027	0.0029	0.0041	0.0022	0.0036	0.0025	0.0031	0.0057	0.0039	0.0025	0.0008	0.0008	0.0006	-0.0012	-0.0025
	p	0.0258	0.0431	0.0064	0.0543	0.0253	0.0418	0.0200	0.0043	0.0098	0.0509	0.2796	0.2483	0.2529	0.6909	0.9205
TH01	Fst	0.0034	0.0029	0.0067	0.0043	0.0025	0.0055	0.0047	0.0015	0.0079	0.0018	0.0058	0.0025	0.0000	-0.0035	-0.0034
	p	0.0710	0.1343	0.0253	0.0649	0.1597	0.0362	0.0472	0.2201	0.0135	0.1792	0.0589	0.1485	0.3683	0.8631	0.8256
TPOX	Fst	0.0081	0.0063	0.0056	0.0060	0.0095	0.0056	0.0083	0.0121	0.0088	0.0046	0.0119	0.0128	0.0073	0.0055	0.0077
	p	0.0155	0.0604	0.0528	0.0433	0.0272	0.0452	0.0199	0.0142	0.0176	0.0812	0.0152	0.0077	0.0245	0.1112	0.0781
VWA	Fst	0.0039	0.0067	0.0031	0.0034	0.0034	0.0060	0.0045	0.0072	0.0060	0.0060	-0.0013	-0.0001	0.0011	-0.0038	-0.0007
	p	0.0398	0.0198	0.0860	0.0671	0.1022	0.0204	0.0342	0.0174	0.0162	0.0243	0.6135	0.4201	0.2247	0.9315	0.4889

Continue Table S10:

Loci		Shanghai-Han	Jiangsu-Huai'an-Han	Jiangsu-Yangzhou-Han	Jiangsu-Taizhou-Han	Anhui-Suzhou-Han	Shangdong-Han	Shanxi-Han	Jiangxi-Jiujiang-Han	Yunnan-Han	Yungui-Han	Taiwan-Han	Xinjiang-Uyghur1	Xinjiang-Uyghur2	Xinjiang-Uyghur3	Xinjiang-Kazakh
CSF1PO	Fst	0.0017	0.0070	-0.0037	0.0019	-0.0005	-0.0017	-0.0017	0.0009	-0.0006	0.0003	-0.0009	-0.0013	-0.0044	-0.0031	-0.0076
	p	0.1893	0.0921	0.8390	0.2677	0.4314	0.5760	0.6994	0.2406	0.5033	0.3274	0.5651	0.6420	0.9805	0.6231	0.9729
D2S1338	Fst	0.0001	-0.0029	-0.0024	-0.0022	0.0012	-0.0004	-0.0002	0.0015	0.0005	0.0003	-0.0001	0.0051	0.0077	0.0046	0.0067
	p	0.3828	0.7977	0.8006	0.6659	0.2786	0.4774	0.4432	0.1414	0.2852	0.3274	0.4406	0.0082	0.0158	0.1229	0.0847
D3S1358	Fst	0.0039	-0.0037	0.0017	0.0053	-0.0012	-0.0015	0.0032	0.0038	0.0035	0.0061	0.0036	0.0081	-0.0007	0.0118	0.0002
	p	0.0865	0.7433	0.2565	0.1491	0.5017	0.5244	0.1185	0.0630	0.0754	0.0251	0.0723	0.0099	0.4484	0.0488	0.3545
D5S818	Fst	-0.0020	-0.0041	-0.0029	-0.0050	-0.0043	-0.0038	-0.0005	-0.0011	-0.0012	-0.0010	-0.0008	0.0014	0.0017	-0.0017	-0.0048
	p	0.8364	0.8566	0.7761	0.8948	0.9714	0.9300	0.4707	0.6619	0.7028	0.6411	0.5708	0.2000	0.2405	0.5269	0.7839

D6S1043	Fst	0.0010	-0.0027	-0.0011	0.0028	-0.0027	0.0013	0.0008	0.0005	0.0000	-0.0002	-0.0008	0.0106	0.0083	0.0033	0.0149
	p	0.2289	0.7938	0.5953	0.1927	0.8801	0.2536	0.2755	0.3012	0.4246	0.4722	0.6838	0.0003	0.0084	0.1690	0.0076
D7S820	Fst	0.0034	0.0079	0.0005	-0.0002	0.0119	0.0073	0.0041	0.0102	0.0079	0.0070	0.0119	0.0048	-0.0007	0.0664	-0.0008
	p	0.0803	0.0594	0.3533	0.4132	0.0097	0.0438	0.0610	0.0013	0.0064	0.0087	0.0008	0.0322	0.4855	0.0000	0.4607
D8S1179	Fst	0.0037	0.0075	0.0061	0.0048	0.0016	0.0045	-0.0003	0.0093	0.0058	0.0091	0.0115	0.0051	0.0039	0.0015	-0.0018
	p	0.0519	0.0459	0.0500	0.1278	0.2442	0.0847	0.4513	0.0001	0.0060	0.0003	0.0000	0.0165	0.1100	0.3021	0.5505
D12S391	Fst	-0.0002	-0.0041	-0.0027	-0.0033	-0.0028	-0.0021	0.0012	0.0007	0.0003	-0.0007	0.0005	0.0024	0.0015	0.0009	0.0112
	p	0.4537	0.9243	0.8211	0.7916	0.8572	0.7302	0.2204	0.2533	0.3346	0.6117	0.2973	0.0847	0.2488	0.3384	0.0385
D13S317	Fst	0.0025	-0.0014	0.0018	-0.0034	-0.0019	0.0043	0.0008	0.0025	0.0015	0.0017	0.0013	0.0057	0.0104	-0.0035	-0.0038
	p	0.1219	0.5450	0.2376	0.7645	0.6588	0.0980	0.2914	0.0888	0.1751	0.1590	0.2026	0.0154	0.0125	0.7388	0.7120
D16S539	Fst	0.0232	0.0266	0.0307	0.0090	0.0312	0.0188	0.0157	0.0199	0.0193	0.0221	0.0215	0.0190	0.0075	0.0078	0.0103
	p	0.0001	0.0002	0.0000	0.0605	0.0000	0.0014	0.0001	0.0000	0.0000	0.0000	0.0000	0.0000	0.0406	0.0808	0.0698
D18S51	Fst	0.0094	0.0015	0.0033	0.0061	0.0016	0.0000	0.0062	0.0083	0.0088	0.0089	0.0166	0.0132	0.0183	0.0180	0.0091
	p	0.0016	0.2763	0.1218	0.0724	0.2283	0.3979	0.0118	0.0006	0.0004	0.0007	0.0000	0.0000	0.0000	0.0030	0.0659
D19S433	Fst	0.0014	0.0001	-0.0004	-0.0020	0.0023	0.0029	0.0003	0.0017	0.0022	0.0016	0.0019	0.0042	0.0064	-0.0019	0.0087
	p	0.1983	0.3919	0.4581	0.6281	0.1810	0.1419	0.3377	0.1262	0.1040	0.1488	0.1147	0.0258	0.0396	0.6094	0.0617
D21S11	Fst	0.0001	-0.0010	0.0000	-0.0042	0.0019	-0.0016	-0.0018	0.0003	0.0006	0.0001	0.0014	0.0005	0.0014	-0.0023	-0.0031
	p	0.3748	0.5159	0.3973	0.8916	0.2067	0.6549	0.8247	0.3396	0.2695	0.3743	0.1670	0.2969	0.2552	0.6568	0.7007
FGA	Fst	-0.0002	-0.0017	0.0003	-0.0019	-0.0036	0.0035	-0.0016	0.0004	-0.0003	0.0000	0.0016	0.0028	0.0034	-0.0025	-0.0005
	p	0.4635	0.6435	0.3810	0.6374	0.9855	0.1045	0.8254	0.3216	0.5056	0.4136	0.1302	0.0610	0.1079	0.7080	0.4603
PentaD	Fst	0.0116	0.0040	0.0116	0.0097	0.0086	0.0044	0.0064	0.0114	0.0097	0.0085	0.0148	0.0029	0.0040	0.0060	0.0033
	p	0.0007	0.1422	0.0084	0.0378	0.0192	0.0907	0.0156	0.0003	0.0002	0.0013	0.0001	0.0734	0.1051	0.1018	0.2220
PentaE	Fst	-0.0011	-0.0013	-0.0032	-0.0028	-0.0021	-0.0007	-0.0017	0.0000	-0.0002	0.0004	0.0009	0.0040	0.0041	0.0010	0.0027
	p	0.7892	0.6538	0.9692	0.8466	0.8661	0.5835	0.9146	0.4523	0.4830	0.3272	0.1850	0.0058	0.0314	0.3062	0.1901
TH01	Fst	-0.0017	-0.0020	-0.0024	0.0000	-0.0026	-0.0013	-0.0023	-0.0006	-0.0011	-0.0011	0.0037	0.0373	0.0490	0.0354	0.0434
	p	0.6986	0.5521	0.6395	0.3630	0.7002	0.4979	0.8214	0.4983	0.6194	0.6025	0.0672	0.0000	0.0000	0.0012	0.0004
TPOX	Fst	0.0072	0.0051	0.0123	0.0002	0.0135	0.0099	0.0141	0.0110	0.0097	0.0157	0.0057	0.0110	0.0101	0.0122	-0.0072
	p	0.0365	0.1461	0.0306	0.3507	0.0214	0.0465	0.0062	0.0055	0.0115	0.0023	0.0438	0.0072	0.0371	0.0577	0.8962
VWA	Fst	-0.0010	-0.0047	-0.0015	0.0007	-0.0013	-0.0011	-0.0012	0.0007	0.0009	0.0034	0.0008	0.0084	0.0078	0.0040	0.0051
	p	0.6034	0.9329	0.5934	0.3519	0.5763	0.5366	0.6334	0.2680	0.2436	0.0587	0.2749	0.0040	0.0317	0.1730	0.1601

Continue Table S10:

Loci		HebeiChengde-Manchu	Liaoning-Hui	Gansu-Hui	Yunnan-Bai	Yunnan-Vietnamese	Yunnan-MiaoS	Yunnan-Yi	Yunnan-Zhuang	Yunnan-Dai	Yunnan-Hani	Yili-Xibe	Liangshan-Yi	Hainan-Han
CSF1PO	Fst	-0.0014	-0.0027	-0.0033	-0.0009	0.0311	0.0394	-0.0009	-0.0017	0.0039	-0.0049	0.0031	0.0079	-0.0043
	p	0.6040	0.7352	0.8433	0.5438	0.0000	0.0000	0.5159	0.5888	0.1801	0.9799	0.1675	0.0597	0.9495
D2S1338	Fst	-0.0003	0.0044	-0.0001	-0.0010	0.0026	0.0083	0.0005	0.0036	0.0049	-0.0033	-0.0021	-0.0022	0.0049
	p	0.4778	0.0657	0.4334	0.7113	0.0985	0.0029	0.3260	0.0858	0.1020	0.9072	0.7861	0.7550	0.0622
D3S1358	Fst	0.0021	-0.0003	0.0085	0.0002	0.0283	0.0263	-0.0014	0.0066	0.0087	0.0098	-0.0038	-0.0042	0.0112
	p	0.1915	0.3966	0.0386	0.3487	0.0000	0.0001	0.6015	0.0698	0.0813	0.0421	0.8973	0.8823	0.0273
D5S818	Fst	-0.0027	-0.0039	-0.0018	-0.0011	0.0011	0.0047	-0.0013	-0.0012	0.0043	-0.0013	-0.0032	-0.0038	-0.0025
	p	0.9143	0.9586	0.6324	0.6346	0.2511	0.0537	0.6444	0.5658	0.1595	0.5416	0.8784	0.8928	0.7358
D6S1043	Fst	-0.0008	-0.0005	0.0018	0.0006	0.0007	0.0019	0.0003	0.0002	0.0027	0.0032	-0.0012	-0.0040	0.0190
	p	0.5933	0.5141	0.2044	0.2966	0.2776	0.1212	0.3512	0.3856	0.1992	0.1276	0.6346	0.9861	0.0000
D7S820	Fst	0.0068	-0.0004	0.0021	0.0032	0.0488	0.0515	0.0058	0.0185	0.0278	0.0070	-0.0009	-0.0030	0.0092
	p	0.0264	0.4368	0.2062	0.0790	0.0000	0.0000	0.0284	0.0006	0.0009	0.0586	0.5104	0.7968	0.0262
D8S1179	Fst	0.0055	0.0020	0.0055	0.0048	0.0539	0.0753	0.0189	0.0232	0.0276	0.0032	0.0076	-0.0001	0.0086
	p	0.0259	0.2087	0.0550	0.0191	0.0000	0.0000	0.0000	0.0000	0.0002	0.1489	0.0214	0.4297	0.0172
D12S391	Fst	-0.0011	-0.0003	-0.0009	-0.0019	0.0281	0.0407	0.0079	0.0091	0.0008	0.0004	0.2191	0.0391	0.0025
	p	0.6422	0.4458	0.5573	0.9226	0.0000	0.0000	0.0044	0.0066	0.3388	0.3682	0.0000	0.0000	0.1678
D13S317	Fst	0.0003	-0.0019	-0.0030	0.0014	0.0434	0.0791	0.0025	0.0099	0.0093	0.0041	-0.0014	-0.0025	0.0009
	p	0.3608	0.6773	0.8703	0.2006	0.0000	0.0000	0.1345	0.0118	0.0432	0.1178	0.5994	0.7416	0.3057
D16S539	Fst	0.0296	0.0223	0.0154	0.0124	0.0499	0.1110	0.0127	0.0088	0.0253	0.0247	0.0183	0.0188	0.0147
	p	0.0000	0.0004	0.0034	0.0010	0.0000	0.0000	0.0012	0.0238	0.0024	0.0004	0.0009	0.0024	0.0053
D18S51	Fst	0.0041	0.0051	0.0095	0.0069	0.0103	0.0151	0.0143	0.0169	0.0123	0.0071	0.0056	0.0017	0.0094
	p	0.0517	0.0543	0.0091	0.0047	0.0008	0.0001	0.0000	0.0001	0.0161	0.0377	0.0455	0.2266	0.0116
D19S433	Fst	0.0008	-0.0024	0.0005	-0.0004	0.0250	0.0335	0.0051	0.0038	-0.0001	0.0025	0.0007	-0.0016	-0.0020
	p	0.2832	0.8083	0.3324	0.5146	0.0000	0.0000	0.0253	0.0897	0.4104	0.1813	0.3197	0.6414	0.7125
D21S11	Fst	0.0018	0.0003	-0.0004	0.0009	0.0014	0.0061	0.0018	-0.0007	-0.0020	0.0002	-0.0022	-0.0030	-0.0021
	p	0.1758	0.3634	0.4624	0.2368	0.2025	0.0104	0.1570	0.5168	0.6424	0.3809	0.7858	0.8842	0.7388
FGA	Fst	-0.0020	-0.0006	-0.0016	0.0015	0.0080	0.0175	0.0110	0.0074	0.0079	0.0105	-0.0020	0.0013	0.0052
	p	0.8800	0.5046	0.7132	0.1460	0.0026	0.0000	0.0002	0.0087	0.0374	0.0064	0.7719	0.2678	0.0429

PentaD	Fst	0.0039	0.0006	0.0031	0.0074	0.0122	0.0162	0.0139	0.0181	0.0339	0.0079	0.0036	0.0069	0.0186
	p	0.0712	0.3413	0.1372	0.0058	0.0012	0.0000	0.0002	0.0002	0.0000	0.0393	0.1169	0.0480	0.0006
PentaE	Fst	-0.0019	-0.0034	-0.0001	0.0020	0.0023	0.0735	0.0024	0.0146	0.0153	0.0078	-0.0004	-0.0017	-0.0015
	p	0.9266	0.9933	0.4498	0.0716	0.0694	0.0000	0.0592	0.0001	0.0002	0.0083	0.5289	0.7645	0.7676
TH01	Fst	-0.0018	-0.0023	0.0019	-0.0024	0.0119	0.0177	0.0037	0.0101	-0.0022	-0.0007	-0.0015	-0.0022	0.0003
	p	0.6712	0.6733	0.2201	0.9358	0.0051	0.0008	0.1034	0.0302	0.5539	0.4395	0.5484	0.6261	0.3446
TPOX	Fst	0.0137	0.0084	0.0017	0.0083	0.1059	0.1190	0.0038	0.0034	0.0043	-0.0051	0.0067	0.0109	0.0047
	p	0.0094	0.0550	0.2431	0.0168	0.0000	0.0000	0.1089	0.1576	0.1844	0.9713	0.0800	0.0405	0.1356
VWA	Fst	-0.0022	-0.0018	0.0016	0.0003	0.0189	0.0321	0.0045	0.0068	0.0011	0.0012	0.0001	-0.0007	0.0043
	p	0.8295	0.6687	0.2496	0.3546	0.0000	0.0000	0.0519	0.0356	0.3297	0.2897	0.4059	0.4724	0.1198

Table S11. The Nei's standard genetic distances between three studied populations and 56 other previously published Chinese populations.

Populations	[01]	[02]	[03]	[04]	[05]	[06]	[07]	[08]	[09]	[10]	[11]	[12]	[13]	[14]	[15]	[16]	[17]	[18]	[19]	[20]
[01] Hainan Han																				
[02] Sichuan Yi	0.0302																			
[03] Sichuan Tibetan	0.0416	0.0234																		
[04] Tibet Tibetan[1]	0.0423	0.0241	0.0272																	
[05] Sichuan Han1[2]	0.0121	0.0163	0.0296	0.0287																
[06] Sichuan Han2[3]	0.0122	0.0135	0.0265	0.0261	0.0018															
[07] Sichuan Han3	0.0161	0.0129	0.0268	0.0285	0.0060	0.0043														
[08] Chongqing Han[4]	0.0137	0.0167	0.0278	0.0282	0.0026	0.0021	0.0048													
[09] Gansu Han[5]	0.0245	0.0171	0.0270	0.0293	0.0111	0.0094	0.0105	0.0093												
[10] Inner Mongolia Han[6]	0.0174	0.0128	0.0254	0.0254	0.0066	0.0047	0.0056	0.0059	0.0096											
[11] Hunan Han[2]	0.0157	0.0154	0.0291	0.0300	0.0035	0.0027	0.0062	0.0036	0.0099	0.0073										
[12] Henan Han1[2]	0.0248	0.0175	0.0290	0.0286	0.0108	0.0095	0.0093	0.0111	0.0135	0.0076	0.0105									
[13] Henan Han2[7]	0.0217	0.0125	0.0237	0.0246	0.0079	0.0063	0.0067	0.0079	0.0106	0.0043	0.0089	0.0088								
[14] Hubei Han1[2]	0.0159	0.0121	0.0246	0.0246	0.0037	0.0020	0.0048	0.0035	0.0097	0.0041	0.0047	0.0074	0.0047							
[15] Hubei Han2[8]	0.0130	0.0130	0.0250	0.0270	0.0024	0.0014	0.0038	0.0029	0.0088	0.0040	0.0029	0.0082	0.0051	0.0018						
[16] Jiangxi Han[2]	0.0141	0.0178	0.0291	0.0297	0.0037	0.0026	0.0063	0.0037	0.0120	0.0072	0.0040	0.0114	0.0082	0.0041	0.0025					
[17] Guangxi Han[2]	0.0149	0.0314	0.0457	0.0451	0.0096	0.0086	0.0173	0.0096	0.0231	0.0177	0.0089	0.0248	0.0209	0.0139	0.0117	0.0100				
[18] Guangdong Han[9]	0.0129	0.0127	0.0242	0.0253	0.0032	0.0016	0.0045	0.0029	0.0089	0.0043	0.0031	0.0095	0.0055	0.0028	0.0019	0.0034	0.0092			
[19] Guangzhou Han[2]	0.0107	0.0204	0.0334	0.0354	0.0045	0.0034	0.0098	0.0048	0.0163	0.0113	0.0044	0.0167	0.0136	0.0070	0.0052	0.0047	0.0033	0.0043		
[20] Dongguan Han[2]	0.0124	0.0235	0.0333	0.0374	0.0067	0.0048	0.0111	0.0064	0.0165	0.0125	0.0064	0.0173	0.0151	0.0082	0.0069	0.0055	0.0047	0.0061	0.0021	
[21] Foshan Han[2]	0.0141	0.0244	0.0379	0.0399	0.0077	0.0065	0.0135	0.0076	0.0197	0.0144	0.0072	0.0213	0.0174	0.0102	0.0085	0.0079	0.0039	0.0065	0.0018	0.0043
[22] Jiangmen Han[2]	0.0113	0.0233	0.0356	0.0372	0.0063	0.0050	0.0119	0.0062	0.0176	0.0132	0.0059	0.0189	0.0162	0.0090	0.0071	0.0059	0.0030	0.0058	0.0013	0.0028
[23] Maoming Han[2]	0.0142	0.0246	0.0390	0.0379	0.0080	0.0060	0.0122	0.0072	0.0181	0.0139	0.0070	0.0188	0.0158	0.0095	0.0077	0.0066	0.0052	0.0062	0.0030	0.0043
[24] Qingyuan Han[2]	0.0126	0.0230	0.0366	0.0382	0.0068	0.0057	0.0113	0.0069	0.0187	0.0133	0.0059	0.0175	0.0158	0.0090	0.0072	0.0065	0.0047	0.0062	0.0018	0.0038
[25] Yangjiang Han[2]	0.0114	0.0233	0.0368	0.0376	0.0051	0.0045	0.0109	0.0053	0.0160	0.0117	0.0053	0.0175	0.0146	0.0080	0.0060	0.0052	0.0032	0.0048	0.0012	0.0028
[26] Zhanjiang Han[2]	0.0122	0.0268	0.0383	0.0410	0.0081	0.0070	0.0145	0.0081	0.0191	0.0146	0.0083	0.0205	0.0172	0.0111	0.0088	0.0087	0.0048	0.0085	0.0030	0.0040
[27] Zhaoqing Han[2]	0.0131	0.0257	0.0382	0.0405	0.0076	0.0060	0.0130	0.0070	0.0188	0.0150	0.0059	0.0206	0.0183	0.0108	0.0084	0.0068	0.0026	0.0066	0.0017	0.0027
[28] Zhongshan Han[2]	0.0132	0.0232	0.0367	0.0386	0.0074	0.0065	0.0130	0.0080	0.0198	0.0142	0.0068	0.0196	0.0179	0.0101	0.0079	0.0066	0.0050	0.0067	0.0019	0.0033
[29] Minnan Han[10]	0.0159	0.0196	0.0326	0.0338	0.0068	0.0055	0.0084	0.0065	0.0143	0.0088	0.0069	0.0139	0.0105	0.0072	0.0061	0.0063	0.0119	0.0063	0.0070	0.0073

[30] Minxi Han[11]	0.0119	0.0168	0.0315	0.0296	0.0035	0.0028	0.0071	0.0041	0.0126	0.0073	0.0033	0.0123	0.0095	0.0048	0.0032	0.0040	0.0070	0.0031	0.0032	0.0052
[31] Xiamen Han[12]	0.0111	0.0145	0.0260	0.0277	0.0026	0.0012	0.0046	0.0026	0.0099	0.0047	0.0031	0.0095	0.0063	0.0024	0.0010	0.0019	0.0095	0.0019	0.0035	0.0048
[32] Beijing Han[13]	0.0241	0.0159	0.0218	0.0283	0.0095	0.0072	0.0098	0.0085	0.0108	0.0074	0.0097	0.0125	0.0075	0.0076	0.0066	0.0089	0.0231	0.0072	0.0151	0.0166
[33] Tianjin Han[14]	0.0180	0.0144	0.0252	0.0256	0.0060	0.0049	0.0058	0.0056	0.0087	0.0043	0.0074	0.0081	0.0051	0.0035	0.0035	0.0059	0.0211	0.0052	0.0124	0.0134
[34] Shanghai Han[15]	0.0161	0.0117	0.0252	0.0260	0.0043	0.0030	0.0040	0.0041	0.0096	0.0050	0.0051	0.0085	0.0053	0.0030	0.0022	0.0042	0.0154	0.0032	0.0076	0.0098
[35] Huaian Han[16]	0.0255	0.0170	0.0292	0.0324	0.0109	0.0091	0.0089	0.0099	0.0140	0.0095	0.0103	0.0138	0.0103	0.0079	0.0076	0.0096	0.0233	0.0083	0.0153	0.0168
[36] Yangzhou Han[16]	0.0215	0.0166	0.0291	0.0276	0.0089	0.0078	0.0090	0.0094	0.0127	0.0072	0.0109	0.0112	0.0089	0.0067	0.0072	0.0087	0.0249	0.0089	0.0154	0.0163
[37] Taizhou Han[16]	0.0309	0.0289	0.0374	0.0350	0.0197	0.0186	0.0235	0.0210	0.0281	0.0211	0.0210	0.0249	0.0209	0.0188	0.0187	0.0193	0.0306	0.0190	0.0229	0.0254
[38] Suzhou Han[16]	0.0235	0.0160	0.0291	0.0279	0.0109	0.0094	0.0089	0.0098	0.0129	0.0070	0.0109	0.0112	0.0078	0.0072	0.0069	0.0103	0.0248	0.0084	0.0170	0.0180
[39] Shandong Han[17]	0.0191	0.0142	0.0273	0.0289	0.0084	0.0076	0.0091	0.0090	0.0138	0.0080	0.0110	0.0136	0.0086	0.0078	0.0072	0.0097	0.0220	0.0081	0.0136	0.0153
[40] Shanxi Han[18]	0.0181	0.0131	0.0212	0.0222	0.0085	0.0064	0.0073	0.0078	0.0098	0.0055	0.0092	0.0088	0.0051	0.0047	0.0054	0.0093	0.0221	0.0060	0.0131	0.0148
[41] Jiujiang Han[19]	0.0118	0.0130	0.0266	0.0275	0.0023	0.0009	0.0039	0.0021	0.0084	0.0038	0.0027	0.0085	0.0055	0.0018	0.0009	0.0021	0.0099	0.0014	0.0038	0.0053
[42] Yunnan Han[20]	0.0123	0.0125	0.0241	0.0262	0.0028	0.0012	0.0039	0.0028	0.0077	0.0039	0.0029	0.0078	0.0050	0.0017	0.0008	0.0025	0.0110	0.0015	0.0048	0.0060
[43] Yungui Han[21]	0.0134	0.0133	0.0271	0.0256	0.0022	0.0011	0.0042	0.0023	0.0093	0.0045	0.0032	0.0087	0.0059	0.0019	0.0019	0.0033	0.0103	0.0022	0.0049	0.0061
[44] Taiwan Han	0.0115	0.0190	0.0301	0.0346	0.0041	0.0034	0.0064	0.0042	0.0129	0.0075	0.0044	0.0129	0.0096	0.0057	0.0036	0.0040	0.0075	0.0036	0.0037	0.0045
[45] Xinjiang Uyghur1[22]	0.0465	0.0367	0.0422	0.0414	0.0365	0.0344	0.0321	0.0360	0.0350	0.0319	0.0331	0.0337	0.0331	0.0337	0.0323	0.0353	0.0432	0.0300	0.0374	0.0387
[46] Xinjiang Uyghur2[23]	0.0587	0.0452	0.0488	0.0498	0.0483	0.0470	0.0455	0.0486	0.0471	0.0434	0.0455	0.0461	0.0415	0.0452	0.0444	0.0486	0.0562	0.0416	0.0503	0.0519
[47] Xinjiang Uyghur3[1]	0.0644	0.0562	0.0635	0.0631	0.0567	0.0549	0.0537	0.0581	0.0579	0.0529	0.0527	0.0542	0.0549	0.0547	0.0537	0.0576	0.0636	0.0504	0.0571	0.0594
[48] Xinjiang Kazakh[24]	0.0688	0.0524	0.0520	0.0602	0.0581	0.0543	0.0534	0.0572	0.0576	0.0506	0.0544	0.0530	0.0530	0.0523	0.0525	0.0562	0.0654	0.0483	0.0565	0.0599
[49] Hebei Manchu[25]	0.0192	0.0123	0.0245	0.0259	0.0070	0.0060	0.0066	0.0071	0.0095	0.0051	0.0081	0.0097	0.0055	0.0046	0.0044	0.0073	0.0214	0.0060	0.0132	0.0139
[50] Liaoning Hui[26]	0.0178	0.0137	0.0224	0.0257	0.0082	0.0074	0.0076	0.0088	0.0122	0.0076	0.0094	0.0116	0.0086	0.0074	0.0063	0.0082	0.0208	0.0059	0.0123	0.0150
[51] Gansu Hui[27]	0.0264	0.0219	0.0264	0.0255	0.0148	0.0142	0.0159	0.0145	0.0160	0.0135	0.0171	0.0185	0.0142	0.0141	0.0134	0.0167	0.0294	0.0136	0.0217	0.0223
[52] Yunnan Bai[28]	0.0160	0.0126	0.0199	0.0225	0.0059	0.0042	0.0069	0.0059	0.0110	0.0065	0.0073	0.0101	0.0068	0.0046	0.0044	0.0069	0.0160	0.0047	0.0089	0.0101
[53] Yunan Vietnamese[29]	0.0629	0.0758	0.1084	0.0789	0.0489	0.0481	0.0564	0.0496	0.0702	0.0583	0.0505	0.0647	0.0619	0.0551	0.0539	0.0517	0.0447	0.0528	0.0502	0.0530
[54] Yunnan Miao[30]	0.0945	0.1100	0.1437	0.1103	0.0781	0.0773	0.0842	0.0787	0.1023	0.0880	0.0817	0.0940	0.0919	0.0850	0.0841	0.0819	0.0775	0.0842	0.0823	0.0840
[55] Yunnan Yi[31]	0.0256	0.0269	0.0392	0.0466	0.0166	0.0151	0.0190	0.0168	0.0249	0.0229	0.0160	0.0273	0.0261	0.0192	0.0176	0.0184	0.0150	0.0148	0.0127	0.0144
[56] Yunan Zhuang[32]	0.0240	0.0408	0.0526	0.0558	0.0190	0.0181	0.0275	0.0206	0.0337	0.0311	0.0190	0.0371	0.0331	0.0251	0.0219	0.0198	0.0087	0.0195	0.0110	0.0124
[57] Yunan Dai[32]	0.0239	0.0404	0.0618	0.0587	0.0191	0.0195	0.0253	0.0190	0.0351	0.0335	0.0187	0.0367	0.0359	0.0242	0.0219	0.0203	0.0133	0.0212	0.0123	0.0161
[58] Yunnan Hani[32]	0.0206	0.0245	0.0359	0.0450	0.0190	0.0164	0.0194	0.0201	0.0263	0.0207	0.0204	0.0282	0.0235	0.0185	0.0173	0.0204	0.0251	0.0176	0.0183	0.0197
[59] Yili Xibe[33]	0.0295	0.0145	0.0235	0.0253	0.0126	0.0116	0.0108	0.0126	0.0159	0.0108	0.0117	0.0139	0.0115	0.0110	0.0104	0.0130	0.0286	0.0104	0.0188	0.0217

Continue Table S11:

Populations	[21]	[22]	[23]	[24]	[25]	[26]	[27]	[28]	[29]	[30]	[31]	[32]	[33]	[34]	[35]	[36]	[37]	[38]	[39]	[40]
[22] Jiangmen Han[2]	0.0028																			
[23] Maoming Han[2]	0.0048	0.0042																		
[24] Qingyuan Han[2]	0.0038	0.0027	0.0034																	
[25] Yangjiang Han[2]	0.0025	0.0018	0.0030	0.0025																
[26] Zhanjiang Han[2]	0.0052	0.0040	0.0040	0.0039	0.0035															
[27] Zhaoqing Han[2]	0.0030	0.0022	0.0029	0.0028	0.0021	0.0035														
[28] Zhongshan Han[2]	0.0032	0.0027	0.0045	0.0034	0.0029	0.0050	0.0036													
[29] Minnan Han[10]	0.0097	0.0084	0.0101	0.0084	0.0076	0.0092	0.0094	0.0103												
[30] Minxi Han[11]	0.0061	0.0046	0.0048	0.0044	0.0041	0.0070	0.0052	0.0056	0.0063											
[31] Xiamen Han[12]	0.0067	0.0050	0.0059	0.0057	0.0042	0.0066	0.0063	0.0062	0.0051	0.0024										
[32] Beijing Han[13]	0.0186	0.0168	0.0174	0.0184	0.0159	0.0191	0.0188	0.0191	0.0119	0.0116	0.0072									
[33] Tianjin Han[14]	0.0169	0.0146	0.0140	0.0146	0.0124	0.0160	0.0161	0.0158	0.0102	0.0083	0.0043	0.0068								
[34] Shanghai Han[15]	0.0117	0.0093	0.0095	0.0091	0.0087	0.0116	0.0111	0.0111	0.0072	0.0056	0.0027	0.0071	0.0044							
[35] Huaian Han[16]	0.0188	0.0169	0.0188	0.0179	0.0168	0.0228	0.0188	0.0190	0.0125	0.0112	0.0082	0.0110	0.0073	0.0074						
[36] Yangzhou Han[16]	0.0214	0.0181	0.0167	0.0177	0.0166	0.0194	0.0194	0.0184	0.0120	0.0109	0.0077	0.0097	0.0056	0.0065	0.0111					
[37] Taizhou Han[16]	0.0282	0.0252	0.0247	0.0242	0.0246	0.0275	0.0277	0.0255	0.0261	0.0214	0.0185	0.0220	0.0218	0.0170	0.0261	0.0221				
[38] Suzhou Han[16]	0.0217	0.0189	0.0189	0.0192	0.0177	0.0223	0.0215	0.0201	0.0151	0.0114	0.0087	0.0098	0.0065	0.0086	0.0106	0.0115	0.0249			
[39] Shandong Han[17]	0.0178	0.0155	0.0173	0.0163	0.0154	0.0169	0.0192	0.0163	0.0122	0.0105	0.0072	0.0087	0.0077	0.0081	0.0126	0.0085	0.0250	0.0111		
[40] Shanxi Han[18]	0.0173	0.0158	0.0159	0.0156	0.0145	0.0158	0.0178	0.0174	0.0114	0.0103	0.0061	0.0076	0.0047	0.0057	0.0110	0.0097	0.0214	0.0082	0.0091	
[41] Jiujiang Han[19]	0.0070	0.0055	0.0065	0.0059	0.0048	0.0077	0.0068	0.0066	0.0050	0.0023	0.0008	0.0068	0.0039	0.0021	0.0071	0.0066	0.0181	0.0073	0.0067	0.0056
[42] Yunnan Han[20]	0.0081	0.0063	0.0070	0.0066	0.0058	0.0082	0.0079	0.0073	0.0058	0.0032	0.0011	0.0065	0.0036	0.0019	0.0076	0.0067	0.0178	0.0070	0.0065	0.0050
[43] Yungui Han[21]	0.0083	0.0066	0.0072	0.0069	0.0057	0.0083	0.0078	0.0081	0.0052	0.0035	0.0020	0.0071	0.0042	0.0034	0.0091	0.0069	0.0190	0.0083	0.0074	0.0059
[44] Taiwan Han	0.0059	0.0047	0.0058	0.0061	0.0039	0.0070	0.0053	0.0063	0.0057	0.0034	0.0023	0.0111	0.0081	0.0053	0.0108	0.0112	0.0231	0.0126	0.0111	0.0103
[45] Xinjiang Uyghur1[22]	0.0379	0.0391	0.0402	0.0378	0.0374	0.0439	0.0387	0.0405	0.0353	0.0346	0.0334	0.0370	0.0345	0.0316	0.0365	0.0398	0.0535	0.0388	0.0426	0.0340
[46] Xinjiang Uyghur2[23]	0.0506	0.0521	0.0543	0.0509	0.0507	0.0587	0.0523	0.0526	0.0497	0.0472	0.0463	0.0492	0.0457	0.0432	0.0463	0.0518	0.0609	0.0471	0.0541	0.0449
[47] Xinjiang Uyghur3[1]	0.0580	0.0594	0.0619	0.0572	0.0581	0.0649	0.0592	0.0586	0.0595	0.0567	0.0558	0.0609	0.0567	0.0543	0.0587	0.0634	0.0723	0.0588	0.0656	0.0539
[48] Xinjiang Kazakh[24]	0.0582	0.0572	0.0596	0.0549	0.0574	0.0659	0.0592	0.0577	0.0605	0.0546	0.0535	0.0562	0.0526	0.0494	0.0557	0.0577	0.0649	0.0591	0.0611	0.0515
[49] Hebei Manchu[25]	0.0183	0.0151	0.0152	0.0156	0.0139	0.0168	0.0172	0.0169	0.0101	0.0087	0.0053	0.0080	0.0046	0.0048	0.0090	0.0069	0.0210	0.0059	0.0079	0.0064
[50] Liaoning Hui[26]	0.0155	0.0145	0.0151	0.0141	0.0128	0.0175	0.0163	0.0153	0.0113	0.0095	0.0071	0.0096	0.0070	0.0071	0.0130	0.0107	0.0232	0.0118	0.0101	0.0077

[51] Gansu Hui[27]	0.0257	0.0229	0.0240	0.0242	0.0217	0.0266	0.0250	0.0252	0.0201	0.0174	0.0145	0.0160	0.0122	0.0128	0.0186	0.0181	0.0280	0.0157	0.0180	0.0138
[52] Yunnan Bai[28]	0.0123	0.0110	0.0117	0.0113	0.0101	0.0123	0.0126	0.0116	0.0110	0.0075	0.0045	0.0092	0.0069	0.0053	0.0119	0.0102	0.0197	0.0116	0.0100	0.0066
[53] Yunan Vietnamese[29]	0.0540	0.0506	0.0515	0.0512	0.0507	0.0546	0.0497	0.0541	0.0509	0.0461	0.0543	0.0632	0.0598	0.0594	0.0618	0.0625	0.0812	0.0574	0.0619	0.0652
[54] Yunnan Miao[30]	0.0881	0.0832	0.0823	0.0833	0.0835	0.0861	0.0814	0.0864	0.0795	0.0762	0.0851	0.0921	0.0873	0.0906	0.0905	0.0891	0.1154	0.0835	0.0891	0.0954
[55] Yunnan Yi[31]	0.0144	0.0156	0.0150	0.0145	0.0135	0.0160	0.0126	0.0135	0.0213	0.0160	0.0170	0.0274	0.0245	0.0203	0.0290	0.0273	0.0388	0.0297	0.0275	0.0260
[56] Yunan Zhuang[32]	0.0115	0.0115	0.0129	0.0122	0.0112	0.0112	0.0098	0.0131	0.0213	0.0175	0.0192	0.0348	0.0340	0.0259	0.0369	0.0358	0.0397	0.0388	0.0339	0.0343
[57] Yunan Dai[32]	0.0129	0.0129	0.0150	0.0127	0.0132	0.0143	0.0130	0.0137	0.0244	0.0178	0.0195	0.0360	0.0325	0.0241	0.0343	0.0372	0.0412	0.0360	0.0339	0.0331
[58] Yunnan Hani[32]	0.0205	0.0201	0.0214	0.0197	0.0185	0.0219	0.0216	0.0214	0.0190	0.0190	0.0162	0.0240	0.0206	0.0192	0.0252	0.0243	0.0387	0.0278	0.0221	0.0228
[59] Yili Xibe[33]	0.0224	0.0212	0.0219	0.0191	0.0204	0.0253	0.0233	0.0217	0.0168	0.0144	0.0122	0.0118	0.0107	0.0093	0.0133	0.0137	0.0246	0.0132	0.0140	0.0111

Continue Table S11:

Populations	[41]	[42]	[43]	[44]	[45]	[46]	[47]	[48]	[49]	[50]	[51]	[52]	[53]	[54]	[55]	[56]	[57]	[58]	[59]
[42] Yunnan Han[20]	0.0006																		
[43] Yungui Han[21]	0.0014	0.0015																	
[44] Taiwan Han	0.0030	0.0034	0.0043																
[45] Xinjiang Uyghur1[22]	0.0331	0.0318	0.0330	0.0318															
[46] Xinjiang Uyghur2[23]	0.0453	0.0439	0.0459	0.0451	0.0100														
[47] Xinjiang Uyghur3[1]	0.0542	0.0539	0.0536	0.0557	0.0272	0.0308													
[48] Xinjiang Kazakh[24]	0.0528	0.0514	0.0551	0.0522	0.0240	0.0248	0.0453												
[49] Hebei Manchu[25]	0.0047	0.0042	0.0050	0.0093	0.0352	0.0451	0.0594	0.0556											
[50] Liaoning Hui[26]	0.0066	0.0062	0.0072	0.0100	0.0270	0.0369	0.0470	0.0437	0.0085										
[51] Gansu Hui[27]	0.0140	0.0132	0.0142	0.0162	0.0283	0.0347	0.0525	0.0474	0.0135	0.0139									
[52] Yunnan Bai[28]	0.0047	0.0039	0.0050	0.0071	0.0312	0.0419	0.0545	0.0480	0.0063	0.0087	0.0141								
[53] Yunan Vietnamese[29]	0.0516	0.0549	0.0477	0.0553	0.1000	0.1217	0.1171	0.1356	0.0626	0.0664	0.0767	0.0642							
[54] Yunnan Miao[30]	0.0815	0.0853	0.0760	0.0859	0.1420	0.1673	0.1578	0.1793	0.0919	0.0992	0.1064	0.0963	0.0096						
[55] Yunnan Yi[31]	0.0167	0.0168	0.0162	0.0154	0.0385	0.0488	0.0592	0.0539	0.0254	0.0217	0.0334	0.0162	0.0657	0.1007					
[56] Yunan Zhuang[32]	0.0205	0.0212	0.0207	0.0170	0.0550	0.0720	0.0776	0.0822	0.0322	0.0323	0.0445	0.0255	0.0548	0.0911	0.0185				
[57] Yunan Dai[32]	0.0201	0.0215	0.0210	0.0186	0.0605	0.0778	0.0822	0.0858	0.0326	0.0328	0.0454	0.0275	0.0579	0.0910	0.0243	0.0167			
[58] Yunnan Hani[32]	0.0173	0.0165	0.0168	0.0165	0.0413	0.0552	0.0673	0.0617	0.0202	0.0212	0.0286	0.0165	0.0775	0.1113	0.0245	0.0305	0.0341		
[59] Yili Xibe[33]	0.0107	0.0103	0.0110	0.0157	0.0273	0.0352	0.0471	0.0417	0.0112	0.0095	0.0185	0.0122	0.0685	0.0998	0.0263	0.0409	0.0410	0.0298	

[1] Z. Wang, D. Zhou, Z. Jia, L. Li, W. Wu, C. Li, et al., Developmental Validation of the Huaxia Platinum System and application in 3 main ethnic groups of China, Sci Rep 6 (2016) 31075.

[2] Q. Liu, Y. Chen, X. Huang, K. Liu, H. Zhao, D. Lu, Population data and mutation rates of 19 STR loci in seven provinces from China based on Goldeneye DNA ID System 20A, Int J Legal Med 131 (2017) 653-656.

[3] G. He, Y. Li, Z. Wang, W. Liang, H. Luo, M. Liao, et al., Genetic diversity of 21 autosomal STR loci in the Han population from Sichuan province, Southwest China, Forensic Sci Int Genet (2017).

[4] X. Zou, Y. Li, P. Li, Q. Nie, T. Wang, Y. Hu, et al., Genetic polymorphisms for 19 autosomal STR loci of Chongqing Han ethnicity and phylogenetic structure exploration among 28 Chinese populations, Int J Legal Med (2017).

[5] W. Zhao, S. Hu, T. Wang, Genetic polymorphisms of 19 STR loci of Pingliang Han of Gansu province, CHIN J FOENSIC MED 30 (2015) 618-619.

[6] J. Wang, Q. Zhong, Z. Zhai, H. Wang, J. Zhang, Y. Wang, Genetic Polymorphisms of 19 STR loci in the Inner Mongolia Han population, Chin J Forensic Med 30 (2015) 82-84.

[7] H. Wang, D. Wu, Z. Feng, Z. Jing, T. Li, Q. Guo, et al., Genetic polymorphisms of 20 short tandem repeat loci from the Han population in Henan, China, Electrophoresis 35 (2014) 1509-1514.

[8] C. Xiao, W. Zhang, T. Wei, C. Pan, D. Huang, Population data of 21 autosomal STR loci in Chinese Han population from Hubei province in Central China, Forensic Sci Int Genet 20 (2016) e13-14.

[9] L. Yang, X. Zhang, L. Zhao, Y. Sun, J. Li, R. Huang, et al., Population data of 23 autosomal STR loci in the Chinese Han population from Guangdong Province in southern China, Int J Legal Med (2017).

[10] Y. Wang, B. Lin, X. Chen, J. Cai, Q. Zhang, Genetic polymorphisms of 20 loci in Han population from Southern Fujian, CHIN J FORENSIC MED 29 (2014) 264-266.

[11] Z. Zhang, T. Chen, Genetic polymorphisms of 20 STR loci in Han population from Western Fujian, CHIN J FOENSIC MED 30 (2015) 411-412.

[12] Y. Lu, P. Song, J.C. Huang, X. Wu, Genetic polymorphisms of 20 autosomal STR loci in 5141 individuals from the Han population of Xiamen, Southeast China, Forensic Sci Int Genet 29 (2017) e31-e32.

[13] X. Ruan, W. Wang, Y. Yang, B. Xie, J. Chen, Y. Liu, et al., Genetic variability and phylogenetic analysis of 39 short tandem repeat loci in Beijing Han population, Yi Chuan 37 (2015) 683-691.

[14] P. Chen, B. Wang, G. He, F. Song, H. Luo, L. Zhang, et al., Population genetic analyses of 20 autosomal STR loci in Chinese Han population from Tianjin in Northern China, Forensic Sci Int Genet 27 (2017) 184-185.

[15] J. Xie, C. Shao, Y. Zhou, W. Zhu, H. Xu, Z. Liu, et al., Genetic distribution on 20 STR loci from the Han population in Shanghai, China, Forensic Sci Int Genet 9 (2014) e30-31.

[16] C. Yin, Q. Ji, K. Li, H. Mu, B. Zhu, J. Yan, et al., Analysis of 19 STR loci reveals genetic characteristic of eastern Chinese Han population, Forensic Sci Int Genet 14 (2015) 108-109.

[17] M.X. Zhang, S.Y. Han, H.M. Gao, S.H. Sun, D.J. Xiao, Y. Liu, et al., Genetic polymorphisms of 19 STR loci in Shandong Han population, Fa Yi Xue Za Zhi 29 (2013) 440-443, 446.

[18] J. Shen, H. Kang, F. Dong, J. Guo, R. Wang, Research of genetic polymorphism on 19 STR loci in Han nationality in Shanxi province and forensic medicine application, Journal of Shanxi Police Academy 23 (2015) 63-66.

[19] J. Yao, K.Y. Xiong, Y. Zhang, Population data of 19 autosomal STR loci in the Chinese Han population from Jiujiang, Southern China, Forensic Sci Int Genet 28 (2017) e47-e48.

[20] L. Huang, X. Chen, C. Xiang, S. Su, Y. Fan, B. Xu, Genetic Polymorphisms of 19 STR Loci in Yunnan Han Population, CHIN J FOENSIC MED 31 (2016) 187-191.

[21] C. Xiang, L. Huang, S. Su, Y. Fan, J. Yu, Y. Huang, et al., Genetic variation of the 20 STRs in Han population from Yunnan-Kweichow plateau of China, Forensic Sci Int Genet 23 (2016) e10-11.

[22] X. Jin, Y. Wei, J. Chen, T. Kong, Y. Mu, Y. Guo, et al., Phylogenic analysis and forensic genetic characterization of Chinese Uyghur group via autosomal multi STR markers, Oncotarget (2017).

[23] G. He, P. Chen, B. Gao, Y. Han, X. Zou, F. Song, et al., Phylogenetic analysis among 27 Chinese populations and genetic polymorphisms of 20 autosomal STR loci in a Chinese Uyghur ethnic minority group, Australian Journal of Forensic Sciences (2017) 1-10.

[24] L. Zhang, C. Xu, H. Chen, Y. Zhou, F. Li, J. Chen, Genetic polymorphism of 19 STR loci in Xinjiang Barkol Kazakh population, Zhong Nan Da Xue Xue Bao Yi Xue Ban 37 (2012) 934-938.

[25] L. Zhao, S. Wu, F. Gu, Y. Zhang, X. Wang, A. Li, Genetic Polymorphisms of 19 STR Loci in Manchu population in Hebei Province, CHIN J FOENSIC MED 31 (2016) 72-73.

[26] H. Shen, S. Yu, F. Guo, J. Yu, P. Jin, Z. Sun, et al., Genetic polymorphisms of 19 STR loci in Hui population in Liaoning province, CHIN J FORENSIC MED 28 (2013) 504-505.

[27] S. Hu, W. Zhao, T. Wang, Genetic polymorphisms of 19 STR loci in Gansu Hui, CHIN J FOENSIC MED 30 (2015) 620-621.

[28] Y. Li, Y. Hong, X. Li, J. Yang, L. Li, Y. Huang, et al., Allele frequency of 19 autosomal STR loci in the Bai population from the southwestern region of mainland China, Electrophoresis 36 (2015) 2498-2503.

[29] X. Zhang, L. Hu, L. Du, A. Nie, M. Rao, J.B. Pang, et al., Genetic polymorphisms of 20 autosomal STR loci in the Vietnamese population from Yunnan Province, Southwest China, Int J Legal Med 131 (2017) 661-662.

[30] X. Zhang, L. Hu, L. Du, A. Nie, M. Rao, J.B. Pang, et al., Genetic analysis of 20 autosomal STR loci in the Miao ethnic group from Yunnan Province, Southwest China, Forensic Sci Int Genet 28 (2017) e28-e29.

[31] X. Zhang, L. Hu, L. Du, H. Zheng, A. Nie, M. Rao, et al., Population data for 20 autosomal STR loci in the Yi ethnic minority from Yunnan Province, Southwest China, Forensic Sci Int Genet 28 (2017) e43-e44.

[32] X. Zhang, L. Du, Z. Huang, T. Gu, L. Hu, S. Nie, Genetic variation of 20 autosomal STR loci in three ethnic groups (Zhuang, Dai and Hani) in the Yunnan province of southwestern China, Forensic Sci Int Genet (2017).

[33] H. Meng, Y. Guo, Q. Dong, G. Yang, J. Yan, J. Shi, et al., Autosomal-STR based genetic structure of Chinese Xibe ethnic group and its relationships to various groups, Int J Legal Med 130 (2016) 1501-1503.

Supplementary Table S12. The detailed information and abbreviations of sample information of all involved populations.

Geographic position	Nationality	Sample size	Abbreviation
Hainan	Han	194	HAH
Sichuan(Liangshan)	Yi	177	SCY
Sichuan(Liangshan)	Tibetan	198	SCT
Tibet	Tibetan	100	TT
Sichuan-1	Han	653	SCH-1
Sichuan-2	Han	2793	SCH-2
Sichuan-3	Han	309	SCH-3
Chongqing	Han	671	CQH
Gansu	Han	217	GSH
Inner Mongolia	Han	426	IMH
Hunan	Han	741	HNH
Henan-1	Han	585	HEH-1
Henan-2	Han	274	HEH-2
Hubei-1	Han	573	HBH-1
Hubei-2	Han	3078	HBH-2
Jiangxi	Han	597	JXH
Guangxi	Han	754	GXH
Guangdong	Han	1533	GDH
Guangdong(Guangzhou)	Han	3940	GZH
Guangdong(Dongguan)	Han	487	DGH
Guangdong(Foshan)	Han	955	FSH
Guangdong(Jiangmen)	Han	1191	JMH
Guangdong(Maoming)	Han	420	MMH
Guangdong(Qingyuan)	Han	1177	QYH
Guangdong(Yangjiang)	Han	1595	YJH
Guangdong(Zhanjiang)	Han	454	ZJH
Guangdong(Zhaoqing)	Han	1283	ZQH
Guangdong(Zhongshan)	Han	687	ZSH
Minnan	Han	351	MNH
Minxi	Han	600	MXH
Xiamen	Han	5141	XMH
Beijing	Han	200	BJH
Tianjin	Han	565	TJH
Shanghai	Han	676	SHH
Jiangsu(Huaian)	Han	137	HUH
Jiangsu(Yangzhou)	Han	184	YZH
Jiangsu(Taizhou)	Han	115	TZH
Anhui(Suzhou)	Han	200	SZH
Shandong	Han	205	SDH
Shanxi	Han	554	SXH
Jiangxi(Jiujiang)	Han	5318	JJH
Yunnan	Han	2384	YNH
Yungui	Han	2490	YGH
Taiwan	Han	775	TWH
Xinjiang-1	Uyghur	1218	XJU-1
Xinjiang-2	Uyghur	214	XJU-2
Xinjiang-3	Uyghur	100	XJU-3
Xinjiang	Kazakh	81	XJK

Hebei(Chengde)	Manchu	423	HBM
Liaoning	Hui	225	LNH
Gansu	Hui	226	GSHU
Yunnan	Bai	1158	YNB
Yunan	Vietnamese	522	YNV
Yunnan	Miao	748	YNM
Yunnan	Yi	559	YNY
Yunan	Zhuang	242	YNZ
Yunan	Dai	116	YND
Yunnan	Hani	170	YNHN
Yili	Xibe	222	YLX
